# Polyphenol-Rich Cranberry Beverage Positively Affected Skin Health, Skin Lipids, Skin Microbiome, Inflammation, and Oxidative Stress in Women in a Randomized Controlled Trial

**DOI:** 10.3390/nu16183126

**Published:** 2024-09-16

**Authors:** Lindsey Christman, Anna De Benedetto, Elizabeth Johnson, Christina Khoo, Liwei Gu

**Affiliations:** 1 Food Science and Human Nutrition Department, Institute of Food and Agricultural Sciences, University of Florida, Gainesville, FL 32611, USA; 2 Department of Dermatology, College of Medicine, University of Florida, Gainesville, FL 32611, USA; 3 Department of Dermatology, University of Rochester Medical Center, Rochester, NY 14620, USA; 4 Ocean Spray Cranberries, Inc., Lakeville, MA 02347, USA

**Keywords:** cranberry, skin aging, skin lipids, skin microbiome, oxidative stress, photoaging

## Abstract

This study aimed to determine whether a polyphenol-rich cranberry beverage affects skin properties, lipids, and the microbiome in women using a randomized, double-blinded, placebo-controlled, cross-over design. Twenty-two women with Fitzpatrick skin types 2–3 were randomized to drink a cranberry beverage or placebo for six weeks. After a 21-day washout, they consumed the opposite beverage for six weeks. Six weeks of cranberry beverage significantly reduced UVB-induced erythema, improved net elasticity on the face and forearm, smoothness on the face, and gross elasticity on the forearm compared to the placebo. When stratified by age, these effects of the cranberry beverage were primarily observed in women >40 years old. SOD activities were improved after six weeks of cranberry beverage consumption compared to the placebo, while glutathione peroxide and TNF-α were improved compared to baseline. These effects were found to differ by age group. Skin lipid composition was modulated by both the cranberry beverage and the placebo. Cranberry beverages did not change α- or β-diversity but altered the abundance of several skin microbes at the species and strain level. Consumption of a cranberry beverage for six weeks improved specific skin properties and oxidative stress and modulated skin lipids and microbiome compared to placebo.

## 1. Introduction

Skin aging is caused by two main mechanisms: intrinsic and extrinsic aging. Intrinsic aging is mainly the result of reactive oxygen species (ROSs) accumulation over time in a natural aging process [1]. Extrinsic aging is due to external factors such as sun exposure, pollution, nutrition, and cigarette smoke. Both mechanisms are associated with oxidative stress, inflammation, and accumulation of advanced glycation end products (AGEs) in the skin tissue.

Human skin microbiota comprises millions of bacteria, fungi, and viruses [2]. The commensal bacteria of the skin protect the human body from pathogens by competing with pathogens for space and nutrients. Skin microorganisms educate the innate and adaptive functions of the cutaneous immune system. Dysbiosis of the skin microbiome has been associated with various cutaneous diseases [3]. The stratum corneum (SC) is the outer layer of skin. SC lipids are produced by keratinocytes and the skin microbiome and consist of ceramides, free fatty acids, and cholesterols [4,5]. The primary function of SC lipids is to provide a barrier against the movement of water and electrolytes and a barrier against microorganism/toxin/allergen invasion. They also modulate the composition of the skin microbiome and signal transduction originating from the epidermal level [6]. Therefore, the skin lipids and microbiome play a role in skin homeostasis. Although the composition of the skin microbiome and lipids has been found to be relatively stable over time, they are susceptible to age-related changes in skin structure and physiology, such as sebum secretion, pH, and hydration [7,8]. However, there is a lack of research on how diet may directly modulate skin lipids and microbiomes.

Due to their anti-inflammatory and antioxidant activity, several studies have examined the effects of polyphenols on skin aging. Dietary intake of green tea polyphenols or a high-flavonol cocoa product for six and 12 weeks was found to contribute to photoprotection and positively affect skin structural characteristics in women aged 40–65 and 18–65, respectively [9,10]. Similarly, pomegranate juice and a polyphenol extract from pomegranate rich in ellagitannins given to women ages 30–45 for 12 weeks were found to increase the UV dose required to cause erythema in the skin [11].

Cranberries (*Vaccinium macrocarpon*) contain a variety of polyphenols, including procyanidins, anthocyanins, flavonols, and phenolic acids [12]. Specifically, cranberry procyanidins contain mostly A-type linkages, unlike most other plant-derived procyanidins with exclusively B-type linkages [13]. Intervention studies have shown that cranberry juice consumption can alleviate plasma oxidative stress and inflammation [14,15]. In vitro, cranberry polyphenols have been shown to have an anti-aging effect by inducing mitophagy in normal human fibroblast cells [16]. In addition, cranberry juice polyphenols have been shown to inhibit collagen glycation and advanced glycation-induced crosslinking in collagen, suggesting an effect on skin aging [17]. Although they have shown antioxidant and anti-inflammatory activity, no clinical trial has tested the effect of cranberry juice on skin photoaging. The primary objective of this trial was to determine whether the daily consumption of a polyphenol-rich cranberry beverage affects aging-related skin parameters, oxidative stress, and inflammation in women aged 25–65. The research on the possible modulations of skin lipids and microbiome by cranberry beverage consumption was exploratory and a secondary objective.

## 2. Materials and Methods

### 2.1. Study Beverages

The polyphenol-rich cranberry beverage was made from a pure high-potency cranberry juice concentrate. The cranberry beverage and placebo were manufactured and packed in 8 oz bottles by Ocean Spray Cranberries (Lakeville—Middleboro, MA, USA). Cranberry beverage contained 192.9 ± 1.5 mg of procyanidins, 19.5 mg anthocyanins, and 24.2 mg of flavonols per bottle, while the placebo did not contain these compounds but had a similar appearance, taste, and color to the test beverage. This dose was determined based on a previous study on the effects of cocoa on skin parameters [10]. The composition of the cranberry beverage and placebo is shown in Supplemental Table S1. Bottles were coded with three-digit numbers by Ocean Spray to ensure double blindness.

### 2.2. Power Analysis

The sample size was calculated using https://sample-size.net/ (accessed on 1 February 2020) based on previous results on the viscoelasticity of the skin after six weeks of a green tea polyphenol beverage [9]. Using a control type I error of α = 0.05 and a type II error of β = 0.2, the calculated sample size was 21. Factoring in a 15% dropout rate, 24 people were enrolled in this study.

### 2.3. Study Design

This randomized, double-blinded, placebo-controlled study was completed at the University of Florida. This study was conducted according to the guidelines of the Declaration of Helsinki and approved by the institutional review board of the University of Florida (IRB201903250, approved on 14 April 2020). All subjects gave consent before beginning the study. The study was registered with https://clinicaltrials.gov (accessed on 1 March 2020) (NCT04183920).

A total of 24 females, 12 aged 25–39 years old and 12 aged 40–65 years old, with Fitzpatrick skin types 2 and 3, were enrolled in this study. These skin types were used to reduce variability among subjects. The exclusion criteria included a BMI of <30 kg/m^2^; pregnancy; breastfeeding; smoking; frequent alcohol use (average >4 drinks/week); a history of skin cancer; intake of medication, such as anti-inflammatories and antibiotics; sunbathing; or the use of tanning beds.

**Figure 1:**
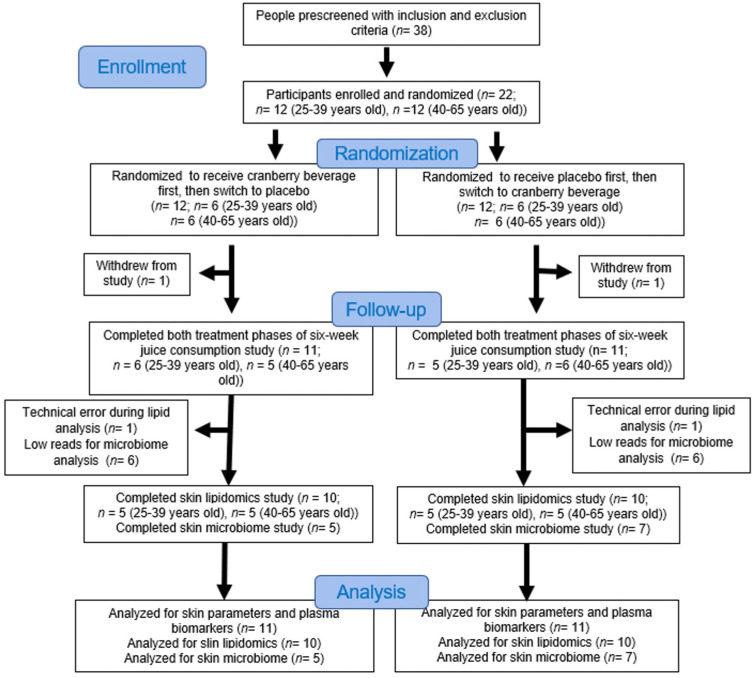
The CONSORT flow diagram of the study shows the enrollment, randomization, participation, and data analysis throughout the study.

The study design is shown in Figure 1 and Figure S1. One week before the start of the study, the individual MED was determined [18]. At this time, subjects were asked to avoid foods rich in procyanidins, including cranberries, blueberries, grapes, chocolate, and plums; botanical dietary supplements; and vitamin and mineral supplements. They were also asked to limit their probiotic yogurts and product intake to less than eight oz per day.

Following this seven-day run-in period, participants were randomly assigned to two groups (*n* = 12 for each group) and asked to consume either two bottles (16 oz) of cranberry beverage or placebo every day for six weeks. A permuted block randomization design, with a block size of four and eight and stratified by age group (<40 years old and ≥40 years old), was implemented for randomization [19]. Bottles were distributed biweekly during the six-week session. After a three-week washout period, the participants switched to the opposite beverage. Two participants dropped out due to scheduling conflicts. Their data were not included in the analysis.

At baseline and the end of the six weeks of each phase, the following variables related to skin parameters were analyzed: sensitivity to UV exposure, skin wrinkles, roughness, scaliness, smoothness, elasticity, hydration, transepidermal water loss (TEWL), pH, color (L*, a*, b*), and melanin and erythema index. Participants were asked to refrain from using any skin care products other than soap 24 h before these skin tests. After washing the skin areas, participants were allowed to acclimate to a room with a temperature of 22.8 ± 0.6 °C and relative humidity of 53.9 ± 1.0% for 10 min. In addition, a fasting blood sample, a skin lipid sample, and a skin microbiome swab were collected.

To monitor if participants were keeping a consistent diet throughout the study, a food frequency questionnaire was filled out at the beginning and end of each six-week session [20]. Several strategies were used to ensure high compliance. Bottles were collected to keep track of how many were consumed by each person. In addition, a questionnaire was collected about how many bottles were missed during sessions. There was 98.7% compliance throughout the study.

### 2.4. Blood Samples

Blood samples were collected after eight-hour fasting at baseline and the end of each intervention period in 10 mL sodium heparin-coated tubes (BD Vacutainer, BD, Franklin Lakes, NJ, USA). The plasma was recovered by centrifuging at 2000× *g* for 10 min at 4 °C. After removing the buffy white film, the remaining blood was washed with isotonic saline and centrifuged to recover erythrocytes for assaying superoxide dismutase (SOD) and glutathione peroxidase (GPx) enzymes. All samples were stored at −80 °C until analysis.

### 2.5. UV-Induced Skin Erythema

Before and after each six-week phase, a 2× minimal erythema dose (MED) was applied to the back using a handheld narrow-band UVB light with a wavelength of 311–312 nm (UV Phototherapy KN4003B, Kernel Medical Equipment, Xuzhou City, China). Skin color was measured using the Skin Colorimeter CL 400 probe (Courage & Khazana, Koln, Germany) before and 24 h after irradiation [18].

### 2.6. Skin Parameter Testing

Skin elasticity was measured using a Cutometer MPA 580 probe (Courage & Khazaka, Koln, Germany) equipped with a two-millimeter measuring probe. Each measurement consisted of a three-second suction at 450 nm of constant negative pressure, followed by a three-second relaxation. The four Cutometer parameters analyzed were gross elasticity (U_a_/U_f_), net elasticity (U_r_/U_e_), viscoelasticity (U_v_/U_e_), and biological elasticity (U_r_/U_f_).

To evaluate the surface characteristics of the skin, a Visioscan VC20+ (Courage & Khazana, Koln, Germany) was used to measure the surface evaluation of living skin parameters. These parameters include roughness, scaliness, wrinkles, and smoothness.

Skin TEWL was measured using a Tewameter TM300 (Courage & Khazana, Koln, Germany), and skin hydration was measured by a Corneometer CM 825 probe (Courage & Khazana, Koln, Germany). Skin pH was measured using a Skin pH meter PH 905 probe (Courage & Khazana, Koln, Germany). Skin color, skin melanin, and erythema index were measured by a Skin Colorimeter CL 400 probe (Courage & Khazana, Koln, Germany) and Mexameter MX 18 probe (Courage & Khazana, Koln, Germany), respectively.

For each skin parameter, two points were measured, one between the middle of the nose and ear and the other between the middle of the wrist and the elbow joint.

### 2.7. Blood Inflammatory and Oxidative Stress Markers

SOD and GPx activities were measured in erythrocytes through colorimetric assays (Caymen Chemicals, Ann Arbor, MI, USA). AGEs were determined in plasma by enzyme-linked immunosorbent assay (ELISA) (Cell Biolab, San Diego, CA, USA). TNF-α and IL-17 were measured by ELISA (Invitrogen, Waltham, MA, USA). High-sensitivity C-reactive protein (Hs-CRP) was determined by ELISA (Advanced Practical Diagnostics, Turnhout, Belgium). All assays were performed following the manufacturer’s instructions.

### 2.8. Skin Lipid Sampling and Analysis

Skin lipids in the stratum corneum were sampled by sequential tape stripping on the forearm using commercially available tapes (22-mm D-Squame discs, CuDerm Corp., Dalla, TX, USA). Four consecutive tape strips were obtained using the D-squame pressure instrument to allow for equal pressure each time. The strips were stored at −80 °C prior to analysis. Additional information on skin lipid extraction and analyses is in Supplementary Methods.

### 2.9. Cutaneous Microbiota Sampling and Analysis

Samples were obtained by swabbing a 4 × 4 cm on the forearm with DNA-free sterile cotton-tipped swabs soaked in 0.9% sodium chloride with 0.1% Tween-20 (Fisher Scientific, Co., Pittsburgh, PA, USA) in a Z-stroke manner [21]. Each sample was collected from the same area. Swabs were stored at −80 °C. Microbiome samples were sent to Cosmo ID for analysis (Rockville, MD, USA). Additional information on microbiome analysis is in the Supplementary Methods.

### 2.10. Shotgun Metagenomic Data Analysis

Metagenomic sequencing reads were analyzed by the Genius bioinformatics software package (https://www.cosmosid.com/, accessed on 22 September 2022) (CosmoID Inc., Rockville, MD, USA) as described elsewhere [22,23]. Sequences with less than 0.5 million reads were not included in the analysis. This is because 0.5 million reads is the lowest sequencing depth to correlate with 2.5 billion reads for species composition by 97% [24]. Additional information on the processing of metagenomic sequence reads is in Supplementary Methods.

### 2.11. Statistics

The 22 participants were separated into two age groups, with 11 in the under-40 group and 11 in the over-40 group. Normal distribution and homogeneity of variance were determined by inspection of standardized residuals. The impacts of treatments, time, and age on skin parameters and blood biomarkers were analyzed with a mixed-effect model. Participants were randomized into two treatment sequences: the placebo-cranberry beverage sequence or the opposite. Possible carryover was tested by contrasting means between six weeks vs. six weeks. The sequence was used as a fixed effect to account for carryover but as a random effect in the absence of carryover. The participants were used as random effects to determine the fixed effects of treatment, time, age, and their interactions. If a main or an interactive effect was significant, a post hoc contrast was used to test the significance level. Changes in skin parameters were calculated by subtracting the baseline value from the six-week value. Changes resulting from cranberry beverages and placebo were compared by post hoc linear contrast.

Lipidomics data was analyzed using multilevel paired multivariate statistics in SIMCA (Version 17.0, Umetrics, Umea, Sweden). All data was unit variance scaled before principal component analysis (PCA) and partial least squares discriminant analysis (PLS-DA). A seven-fold cross-validation was used to evaluate the model and was confirmed by analysis of variance of cross-validated residuals (CV-ANOVA). The robustness of each model was analyzed by a 200-cycle permutation test. The discriminant lipids were determined by variable importance in projection (VIP) value ≥1.50.

Alpha diversity and β diversity were calculated from the relative abundance matrix of family, genus, and species level from the Cosmo ID taxonomic analysis (https://www.cosmosid.com/, accessed on 22 September 2022). Diversity measures were calculated using the Genius bioinformatics software package (https://www.cosmosid.com/, accessed on 22 September 2022). Statistical significance and plot visualization were performed in R. Wilcoxon rank sum tests were performed to determine the significance for Chao1 and Shannon index. Permutational multivariate analysis of variance (PERMANOVA) tests for each distance matrix were generated. To determine the effects of cranberry beverage consumption on the skin microbiome and the association with skin parameters, the relative abundance of each taxon was analyzed using the MaAsLin2 R package v1.18.0 (microbiome multivariate association with linear models) [25]. Additional information on MaAslin2 analysis can be found in Supplemental Methods.

## 3. Results

### 3.1. Characteristics of Subjects at Baseline

Twenty-four participants were enrolled and randomized at the beginning of this study. Two participants dropped out due to scheduling issues after the study’s first phase. Twenty-two participants completed both treatment phases in the six-week study (Figure 1). Data from these 22 participants were included in the skin parameters and blood testing results. The characteristics of the participants, including their age, weight, BMI, Fitzpatrick skin type, and MED, are presented in Table S2. There were no significant differences between groups at baseline (*p* > 0.05).

Twenty participants were included in the skin lipid analysis due to a technical error in the lipid extraction of two samples. Human skin has a very low bacterial load compared to gut and mucosa, which often results in low sample reads. After excluding samples with low reads, twelve participants were included in the skin microbiome analysis. Ten participants were included in the microbiome–lipid correlation analysis. The food frequency questionnaire results confirmed that participants did not change their habitual diet throughout the study (Table S3).

### 3.2. Cranberry Beverage Protected against UV Irradiation

The effect of cranberry beverage on erythema was measured as the difference of a colorimeter a*-value taken before and 24 h after UV irradiation exposure at 2× MED (Table 1). Six weeks of cranberry beverage significantly reduced UV-induced erythema compared to placebo and baseline. The change in erythema from 6 weeks to baseline (∆a* 6 week—∆a* baseline) was significantly lower after drinking cranberry beverages in comparison to placebo (*p* ≤ 0.05). When stratified by age, these effects were only seen in women ≥40 years old but not in women <40 (Table S4).

### 3.3. Cranberry Beverage Improved Net Elasticity and Smoothness on the Face

After drinking cranberry beverages for six weeks, participants had improved net elasticity and smoothness on their faces based on the positive changes from the baseline compared to the placebo in all participants (*p* ≤ 0.05) (Table 2). After stratification by age groups, the improvement in net elasticity was only observed in women over 40 (Table S5). The smoothness improvement was not observed in either women over or below 40, likely due to decreased statistical power after stratification. Cranberry beverage consumption significantly decreased TEWL but increased biological elasticity in all participants and women ≥40 years old compared to baseline. Improvement in gross elasticity and wrinkles compared to baseline was only observed in women ≥40 years old in the cranberry beverage group. The decrease in wrinkles after cranberry beverage consumption was detected by analyzing Visioscan images (Figure S2). Cranberry beverage increased face net elasticity compared to placebo and baseline in all participants and in women ≥40 years old but not in those <40. Other skin parameters, including TEWL, hydration, pH, color, roughness, or scaliness, were not affected by the cranberry beverage (Table 2 and Table S5).

### 3.4. Cranberry Beverage Improved Gross Elasticity and Net Elasticity on the Forearm

A greater change from baseline at six weeks of cranberry beverage consumption showed an improved gross elasticity and net elasticity on the forearm in all participants and women ≥40, but not in women <40 (Table 3 and Table S6). In women ≥40 years old, the cranberry beverage increased gross and net elasticity compared to placebo and baseline. However, this was not observed in women <40. The cranberry beverage increased biological elasticity compared to baseline in all participants and women ≥40 years old, but not in those <40. This was accompanied by a decrease in viscoelasticity after six weeks of cranberry beverage compared to baseline. Other skin parameters on the forearm were not affected by cranberry beverage intake.

### 3.5. Cranberry Beverage Modulated Inflammatory and Oxidative Stress Biomarkers

**Figure 2:**
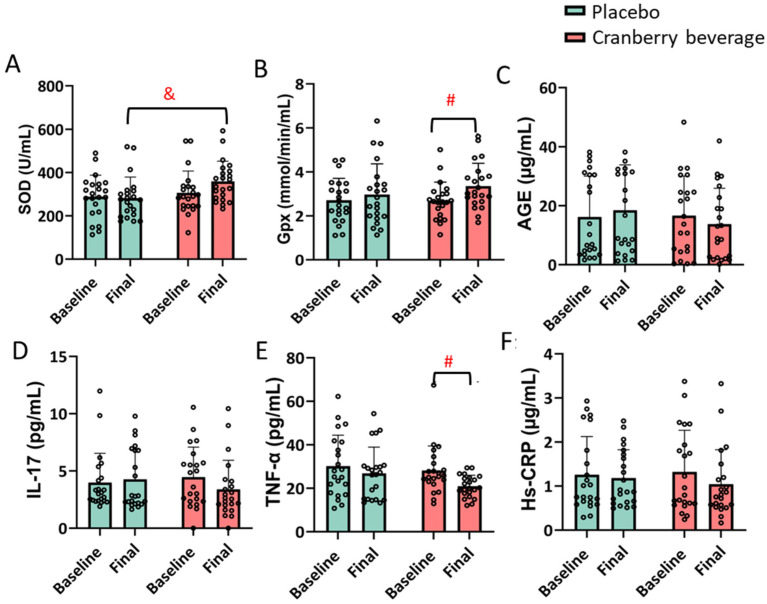
Plasma levels of SOD (**A**), GPx (**B**), AGE (**C**), TNF-α (**D**), IL-17 (**E**), and Hs-CRP (**F**) in all 22 participants. Plasma was collected at baseline and final time points after six weeks of cranberry beverage or placebo consumption. Results are expressed as mean ± SD. ^&^ is the significant difference between cranberry and placebo; ^#^ is the significant difference between final and baseline. SOD: superoxide dismutase, GPx: glutathione peroxide; IL-interleukin; TNF: tumor necrosis factor; Hs-CRP: high-sensitivity C-reactive protein.

Cranberry beverage consumption for six weeks increased the activity of SOD compared to placebo. An increased GPx activity and decreased TNF-α levels were observed after cranberry beverage consumption compared to the baseline but not to the placebo in all participants (*p* ≤ 0.05) (Figure 2). When stratified by age, the effects on SOD and TNF-α were significant in women <40 years old but not in those ≥40 years old (Figure S3). The opposite was observed for GPx activity. The IL-17, CRP, or AGE levels were not affected.

### 3.6. Multivariate Analysis of Skin Lipids

The lipidomic profile between groups was compared using both unsupervised PCA and supervised paired PLS-DA methods. The PCA plot in Figure S4 shows little separation between groups in all participants.

**Figure 3:**
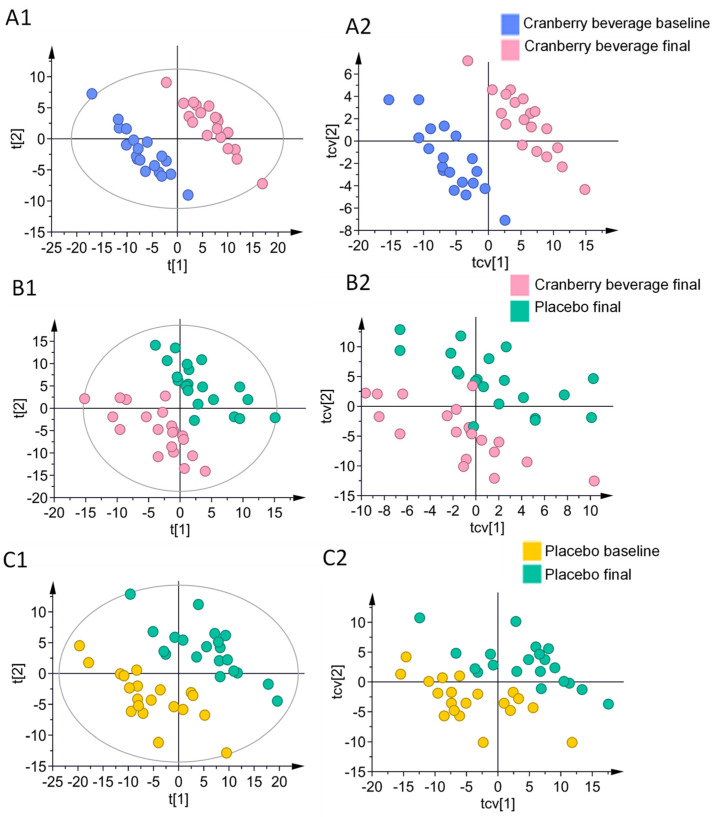
Cranberry beverage intake altered skin lipids compared to baseline and placebo. Panels (**A1**,**B1**,**C1**) are score plots derived from paired partial least squares discriminant analysis (PLS-DA). Panels (**A2**,**B2**,**C2**) are corresponding cross-validated score plots. Each dot represents a participant (*n* = 20).

The segregation of groups in the PLS-DA score plots showed that cranberry beverage consumption altered the skin lipidomic compared to both baseline and the placebo (Figure 3A1,B1). Separation between placebo baseline and post-placebo consumption was also observed (Figure 3C1). The corresponding cross-validation plots showed a similar separation between groups. Table S7 summarizes parameters for PLS-DA models built between groups. All models in the multilevel paired analysis exhibited good predictability (Q2 < 0.5, R2Y < 0.8) and were confirmed to be valid by CV-ANOVA and permutation test (CV-ANOVA *p*-value ≤ 0.05, R2 intercept < 0.5, Q2 intercept < 0.05) (Figure S5).

### 3.7. Discriminant Skin Lipids after Cranberry Beverage and Placebo Consumption

Discriminant lipids were selected by combining the results of paired PLS-DA models (VIP ≥ 1.50) and univariate analysis (*p*-value ≤ 0.05 by paired *t*-test). In metabolomic studies, discriminant metabolites are typically identified using a VIP value cutoff of 1.00 or 1.50 as a sole criterion. We chose to add univariate analysis as an additional criterion to enhance their validity. A total of 12 lipids were found to be discriminant between baseline and after cranberry beverage consumption (Table 4). Seven discriminant lipids were found between the baseline and placebo final. When comparing post-cranberry beverage and post-placebo consumption, the levels of an NDS [DS(C20)26:0] and two EOS ceramides [S(C21)w32:0_18:2 and S(C22)w30:0_18:2] were higher after cranberry beverage consumption, while the level of an NP ceramide [P(18)29:0] was significantly higher after placebo consumption.

### 3.8. Discriminant Skin Lipids Correlated with Some Skin Parameters

**Figure 4:**
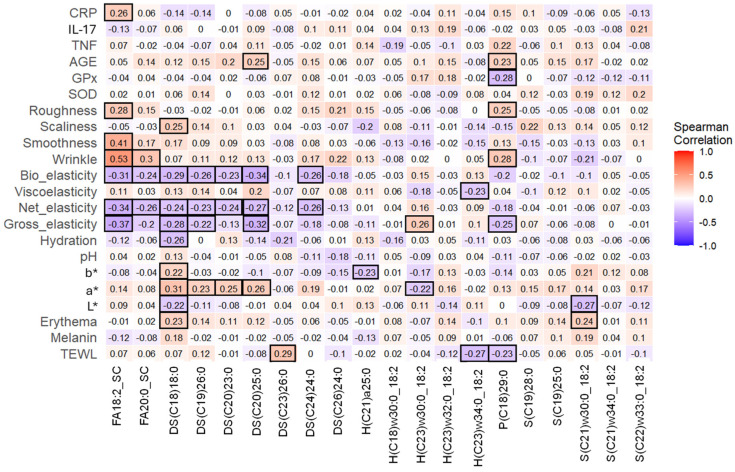
Spearman correlation of discriminant skin lipids, skin parameters, and blood biomarkers for all participants (*n* = 20). Different colors represent differences in Rho value. The bold black outlines indicate significant correlations.

Spearman correlation analysis assessed the relationship between discriminant lipids, skin parameters, and inflammatory and oxidative stress biomarkers (Figure 4). DS(C18)18:0 positively correlated with the color values a*, b*, and the erythema value, but negatively correlated with L* (*p* ≤ 0.05). Four additional ceramides were correlated with a*. Five ceramides and two fatty acids negatively correlated with biological elasticity and net elasticity, while four of these, including FA18:2_SC, DS(C18)18:0, DS(C19)26:0, DS(C20)25:0, and negatively correlated with gross elasticity (*p* ≤ 0.05). In addition, H(C23)w32:0_18:2 positively correlated but P(C18)29:0 negatively correlated with gross elasticity. Wrinkles positively correlated with FA18:2_SC, FA20:0_SC, and P(C18)29:0. Smoothness and roughness positively correlated with a fatty acid, and scaliness was positively correlated with DS(C18)18:0 (*p* ≤ 0.05). TEWL positively correlated with SC(C23)26:0 and negatively correlated with H(C23)34:0_18:2 and P(C18)29:0. For the inflammatory and oxidative stress biomarkers, AGE positively correlated, whereas GPx negatively correlated with P(C18)29:0 (*p* ≤ 0.05). In addition, AGE positively correlated with DS(C20)25:0 and CRP positively correlated with FA18:2_SC.

### 3.9. Cranberry Beverage Did Not Affect Skin Microbial Diversity

Cranberry beverage or placebo did not affect α-diversity on the forearm measured by Chao1, Shannon index, or Simpson index at the species level (Figure S6). Beta-diversity was measured using Bray–Curtis dissimilarity and Jaccard similarity and was visualized by principle coordinate analysis (PCoA) (Figure S6). There was no difference among groups as measured by a permutation ANOVA test for Bray–Curtis (*p* = 0.398) or Jaccard distance (*p* = 0.706).

### 3.10. Cranberry Beverage Modulated Skin Microbiota Composition at Species and Strain Level

Microbiome analysis showed that at the phylum level, the skin bacterial composition was primarily made of *Actinobacterium*, *Firmicutes*, and *Proteobacteria* (Figure S7). At the genus level, the top taxa were *Micrococcus*, *Cutibacterium*, *Propionibacterium*, *Cellulosimicrobium*, *Staphylococcus*, and *Dermacoccus*.

**Figure 5:**
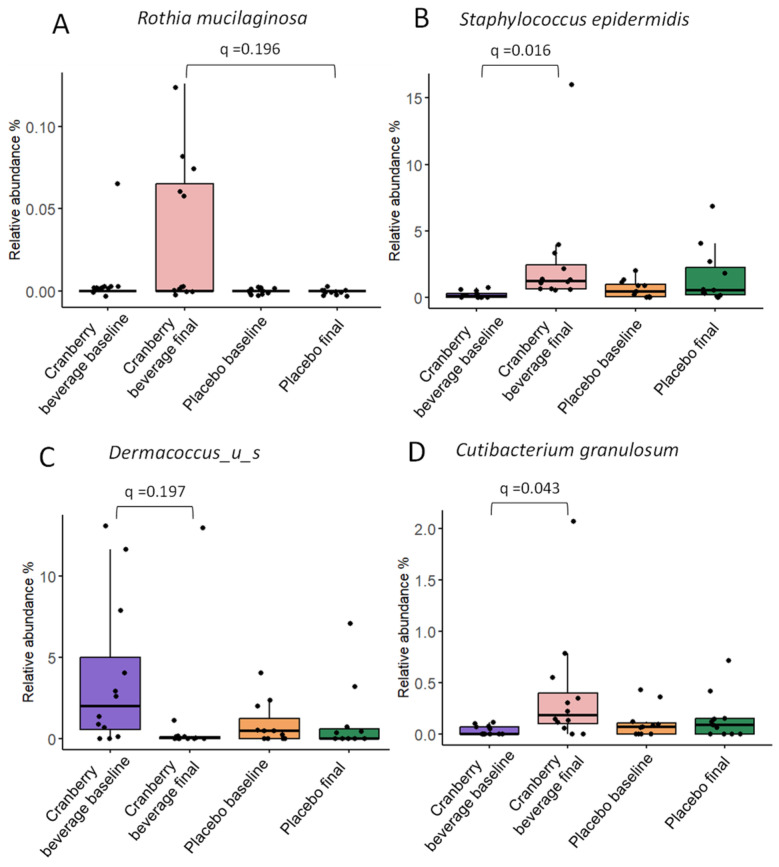
Cranberry beverage intake affected the relative abundance of differential taxa at the species level. The *q* values were calculated by MaAsLin2. The boxes represent the interquartile range (IQR) between the first and third quartiles. Dots indicate individual participants.

**Figure 6:**
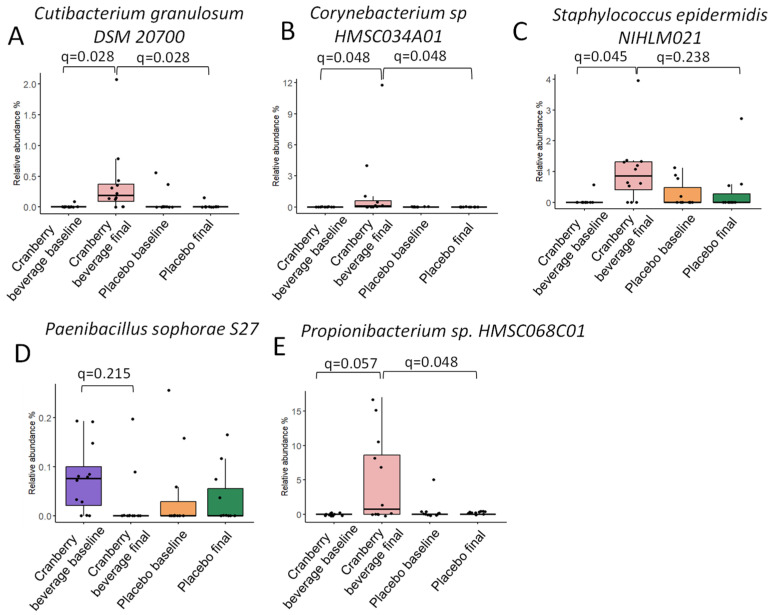
Cranberry beverage intake affected the relative abundance of differential taxa at the strain level. The q values were calculated by MaAsLin2. The boxes represent the interquartile range (IQR) between the first and third quartiles. Dots indicate individual participants.

Multivariate linear regression modeling, MaAsLin2, was used to determine taxa that were associated with cranberry beverage or placebo consumption. Four species and four strains differed between groups (Figure 5 and Figure 6). At the species level, six weeks of cranberry beverage resulted in a higher abundance of *Rothia mucilaginosa* compared to placebo (Figure 5A). Cranberry beverage increased the abundance of *Cutibacterium granulosum* and *Staphylococcus epidermidis* but decreased an unknown *Dermacoccus* species compared to baseline (*q* ≤ 0.25). At the strain level, participants who consumed cranberry beverages were found to have a significantly higher abundance of *Cutibacterium granulosum DSM 20700, Propionibacterium* sp. *HMSC068C01*, and *Corynebacterium* sp. *HMSC034A01* compared to placebo and baseline (Figure 6A,B). The abundance of *Staphylococcus epidermidis NIHLM021* was increased, but *Paenibacillus sophorae S27* was decreased by cranberry beverage consumption compared to the baseline (*q* ≤ 0.25) (Figure 6C,D).

### 3.11. Skin Microbiota Correlated with Skin Parameters

**Figure 7:**
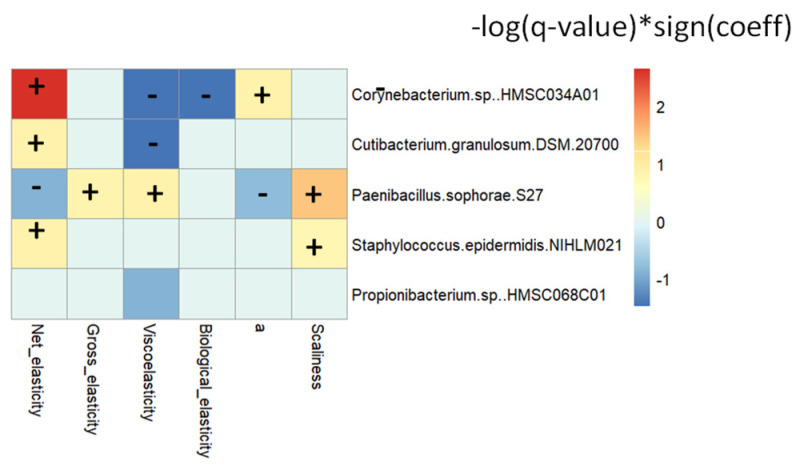
Significantly changed microbial taxa correlated with skin parameters, oxidative stress, and inflammatory biomarkers. Significant correlations were identified using MaAsLin2 and are plotted as (−log(*q*-value) * sign(coeff)). Significant positive or negative correlations were indicated by ‘+’ and ‘−‘.

Associations between forearm skin parameters and discriminant skin species and strains were examined using MaAsLin2. The results are displayed in Figure 7. *Cutibacterium granulosum DSM 20700*, *Staphylococcus epidermidis NIHLM021*, and *Paenibacillus sophorae S27* were positively or negatively associated with elasticity (*q* ≤ 0.25). *Paenibacillus sophorae S27* and *Corynebacterium* sp. *HMSC034A01* were associated with the color value a* (*q* ≤ 0.25), while *Paenibacillus sophorae S27* and *Staphylococcus epidermidis NIHLM021* were positively associated with scaliness.

### 3.12. Skin Microbiota Correlated with Discriminant Skin Lipids

**Figure 8:**
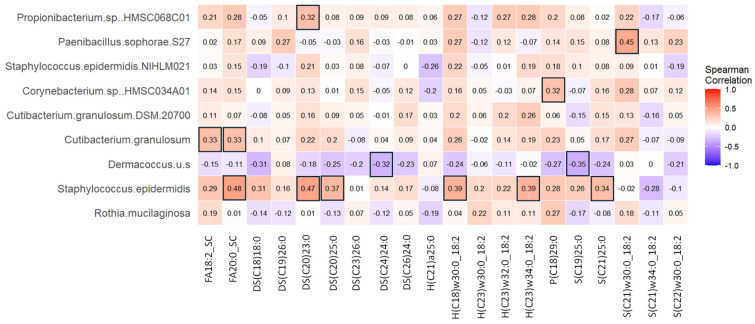
Spearman correlations between discriminant skin lipids determined by paired PLS-DA and significantly changed microbial taxa determined by MaAsLin2. Different color values represent different Rho values. The bold black outline indicates significant correlations.

Correlations between discriminant lipids and microbial taxa significantly changed by cranberry beverage consumption were analyzed by Spearman correlation analysis (Figure 8). *Staphylococcus epidermidis* was found to correlate positively with several lipids, including FA20:0_SC, DS(C20)23:0, DS(C20)25:0, H(C18)w30:0_18:2, H(C18)w34:0_18:2, S(21)25:0 (*r* > 0.30, *p* < 0.05). An unknown *Dermacoccus* species negatively correlated with DS(C24)24:0 and S(C19)25:0 (*r* < −0.3, *p* < 0.5). *Propionibacterium* sp. HMSC068C01 positively correlated with DS(C20)23:0, *Cutibacterium granulosum* positively correlated with two fatty acids, and *Paenibaccilus sophorae S27* positively correlated with S(C21)w30:0_18:2 (*r* > 0.3, *p* < 0.05).

## 4. Discussion

In this randomized, double-blinded, placebo-controlled, cross-over study, we found that a polyphenol-rich cranberry beverage positively affected some age-related skin parameters, mostly in women ≥40 years old. Age appeared to be the most prominent factor affecting skin parameters. Women ≥40 years old were more responsive to dietary intervention than their younger counterparts. The skin microbiome composition at the species and strain level was shifted after drinking cranberry beverages for 6 weeks. Cranberry beverages caused significant changes in skin lipid composition compared to both baseline and placebo, suggesting a systemic modulation of skin homeostasis by diet.

UV irradiation is a predominant factor in skin aging. Acute exposure to UV induces free radical production and inflammatory reactions in the skin. This is characterized by vasodilation and increased blood flow to the dermis, resulting in sunburn erythema. Six weeks of cranberry beverage consumption protected skin from UV-induced erythema in all participants and women ≥40 years old. Similar results have been reported for green tea catechin, cocoa flavonols, pomegranate juice, apple polyphenols, carotenoids, and lycopene [9,10,11,26,27,28]. The antioxidant and anti-inflammatory activities of polyphenols were thought to be the major mechanism of UV protection. UV protection was only observed in older participants, likely because they had elevated oxidative stress and inflammation compared to younger ones.

Cranberry beverage improved net elasticity on the face and forearm as well as gross elasticity on the forearm. When stratified by age, this effect was seen in women >40 years old but not in younger women. Skin elasticity has been shown to decrease with age, which may make it more amenable to dietary intervention [26,27]. Collagen plays a crucial role in maintaining skin elasticity because it provides scaffolding support for the skin. Collagen production naturally declines with age, starting in the mid-20s, causing a decrease in skin elasticity and firmness, contributing to wrinkles and sagging skin formation. UV exposure leads to the upregulation of MMPs that degrade collagen and elastin fibers, contributing to the loss of collagen [1,29]. Additionally, collagen fibers in the human skin are susceptible to glycation due to their long half-life of about 15 years. The formation of AGEs and the subsequent AGE-induced crosslinking cause collagen degradation and function loss. Our previous research suggested that procyanidins, flavonols, and anthocyanins in cranberry juices protected skin collagen by inhibiting glycation caused by methylglyoxal and dehydroascorbic acid [17]. Polyphenols were also reported to preserve skin collagens by regulating MMP and type I procollagen production [30]. The improvement of skin elasticity was consistent with the increase in face smoothness in all participants because this is closely related to collagen. The decrease in viscoelasticity ratio in the cranberry beverage group compared to the baseline is aligned with the results for gross, net, and biological elasticity, as this is a measure of the stiffness of the elastin fibers and has been reported to increase with age [31]. Six weeks of cranberry beverage positively affected face TEWL and forearm biological elasticity in all participants and women ≥40 years old compared to baseline but not to the placebo. However, no effects of the cranberry beverage intake were observed for the majority of skin parameters, including hydration, pH, color, viscoelasticity, roughness, and scaliness. Similar results were reported in a randomized controlled trial for the effects of avocado consumption on skin parameters in women aged 27–73 years [32]. This may be explained by the dose and duration of the cranberry beverage intake and the small sample size of women in this trial. Another possible reason is the variation in skin hydration, pH, and scaliness with temperature, humidity, air pressure, sun exposure, and precipitation [33].

Overall, skin health parameters in older women were much more responsive to polyphenol-rich cranberry beverages than their younger counterparts. A similar trial found that oleocanthal and oleacein serum formulation for 30 days reduced wrinkles in women aged 45–79 but not in women aged 20–44 [34]. This was likely because older women have declined skin health and antioxidant functions due to aging and thus can benefit more from dietary intake of polyphenols.

Six weeks of cranberry beverage consumption increased the activity of SOD in the red blood cells in all participants. SOD plays a key role in protecting skin from photoaging because it neutralizes superoxide radicals and promotes collagen production through the activation of AMPK and Nrf2/HO-1 cascades [35]. Contradicting the observations on skin parameters, an improvement in SOD was found in women <40 but not in those ≥40. This is likely because many other factors affect SOD activity, including genetics, age, and physical activities. It may also suggest that cranberry beverages affect skin health through antioxidant-independent pathways. Cranberry beverage was also found to decrease TNF-α compared to baseline but not compared to placebo. TNF-α is a pro-inflammatory cytokine that is central to the inflammatory response. TNF-α is a key cytokine involved in aging and the pro-inflammatory process of the skin by inhibiting collagen synthesis and inducing increased production of MMP-9 [36]. Cranberry beverage did not affect IL-17, HS-CRP, or AGE. Previous studies on the effect of cranberry beverages on inflammation and oxidative stress also found mixed results [15,37].

Skin lipids are produced by keratinocytes and the skin microbiome and consist of ceramides, free fatty acids, and cholesterols [5]. The primary function of epidermal lipids is to provide a barrier against the movement of water and electrolytes and a barrier against microorganism invasion. They also modulate the composition of the skin microbiome and signal transduction originating from the epidermal level [6]. The lipid composition of the stratum corneum differed between placebo and cranberry beverage 6 weeks post-consumption. Both placebo and cranberry beverages modulated skin lipids compared to baseline, indicating that both treatment and time affected skin lipids. Cutaneous lipidomics are known to be affected by environmental factors, psychological stress, and hormonal changes [38,39,40]. However, there is very little research on how diet and nutrition directly affect skin lipidomics.

Eighteen out of twenty discriminant skin lipids were ceramides (Table 4). Ceramide accounts for about 50% of epidermal lipids and plays a critical role in maintaining the skin’s barrier function and hydration levels. Abnormal ceramide composition in the skin has been implicated in the development of atopic dermatitis and psoriasis. The correction between ceramides and skin parameters, including inflammation biomarkers, suggested that cranberry beverages affected skin health through direct modulating of ceramide biosynthesis and degradation (Figure 4).

Cranberry juice did not affect the alpha or beta diversity; however, there were changes in the abundance of several microbes at the strain and species level. Similar observations were made on the effects of cranberry beverages on the human gut microbiome. Twenty-four weeks of cranberry beverage was found to decrease the abundance of an unnamed *Flavonifractor* species without affecting overall taxonomic composition and diversity in women [41]. Two weeks of high-polyphenol cranberry beverage increased fecal abundance of *Faecalibacterium prausnitzii* and *Eggerthella lenta* in obese adults, whereas overall diversity was not affected [42]. The studies suggested the effect of cranberry on the human gut or skin microbiome may be strain-specific.

Cranberry juice consumption increased the relative abundance of *Rothia mucilaginosa* at the species level, *Cutibacterium granulosum DSM 20700, Corynebacterium* sp. *HMSC034A01,* and *Propionibacterium* sp. HMSC068C01 at the strain levels compared to placebo (Figure 6 and Figure 7). *Rothia mucilaginosa* and *Corynebacterium* sp. are commensal bacteria in human skin and mucosa. *Cutibacterium granulosum* is associated with stimulation of the immune system and has been found to have beneficial effects on the skin barrier by preventing the colonization of pathogenic bacteria [43,44]. *Propionibacterium* is a commensal residential skin microbiota and helps break down sebum, producing fatty acids that contribute to maintaining the acidic pH of the skin [45]. Species of *Propionibacterium* may protect against certain infections by outcompeting pathogens or by positively stimulating the immune system [46]. Cranberry beverage increased the abundance of *Staphylococcus epidermidis* compared to baseline but not to placebo. *Staphylococcus epidermidis* is a commensal bacterium commonly found on human skin. It is often associated with opportunistic infections when the skin barrier is compromised but also serves several important functions in maintaining skin health [47,48]. The correlation between these skin microbiomes and skin parameters, including inflammation biomarkers, suggests a direct impact of skin microbes on skin homeostasis (Figure 7).

*Staphylococcus epidermidis* was found to be positively correlated with several ceramides, likely because of their reported ability to secrete an enzyme involved in ceramide synthesis and lipid metabolism (Figure 8) [4,7].

A strength of this study was the enrollment of equal numbers of women below and above 40 years old, which helped to reveal that skin parameters in women ≥40 were more responsive to the dietary intervention. Another strength of this study was the identification of discriminant skin lipids and microbes that correlated with skin parameters and aging biomarkers, suggesting their essential role in maintaining skin homeostasis.

A limitation of this study was the relatively small size of women ≥40 years old. A future study with a larger sample size of participants ≥40 years old is needed to confirm these results. Some measurements that were significantly changed in the whole population were no longer significant when stratified by age, most likely due to the decreased power in these subgroups. Another limitation of this study was that due to the low biomass of microbes on the skin, the read count of several samples was very low, leading to few people being included in the microbiome analysis. There is a large interpersonal variation in the bacterial composition of the skin, so a future larger study is needed to confirm these results because a sample size below the calculated power may lead to overinterpretation of the findings [49]. The existence of carryover in part of the skin parameters in the cross-over design is another limitation, even though it was accounted for in the mixed linear model.

## 5. Conclusions

In conclusion, six weeks of polyphenol-rich cranberry beverage consumption protected skin against UV-induced erythema, improved skin elasticity, and reduced oxidative stress. Most of the skin parameter benefits were observed in women ≥40 years old, whereas younger women were much less responsive. Skin lipids were found to be modulated after both cranberry beverage and placebo consumption, while the skin microbiome was altered at the species and strain level by cranberry beverage consumption. These findings suggest a potential benefit of cranberry beverages on skin health in women ≥40 years old and warrant future studies on this group of women and the mechanism behind this.

## Figures and Tables

**Table 1:** Change in skin redness (∆a*) caused by UVB irradiation (2× MED) before and after 6 weeks of cranberry beverage or placebo consumption.

	Time Period	Placebo	Placebo Change from Baseline	Cranberry Beverage	Cranberry Change from Baseline
∆a*	Baseline	8.8 ± 2.9	0.003 ± 3.2	9.3 ± 3.1	−2.1 ± 2.8 ^&^
	6 weeks	8.8 ± 3.1		7.2 ± 3.5 ^#,^^	

Data are expressed as mean ± SD (*n* = 22) and organized by time and treatment. Change from baseline = ∆a* 6 week—∆a* Baseline; ^#^ is the significant difference between baseline and after six weeks in the same treatment groups by post hoc contrast; ^ is the significant difference between cranberry beverage and placebo at the same time point by post hoc contrast; ^&^ is the significant difference between cranberry beverage and placebo by post hoc linear contrast.

**Table 2:** Skin parameters on the face in women who consumed cranberry beverage or placebo for six weeks.

Skin Parameters	Time Period	Placebo	Change from Baseline	Cranberry Beverage	Change from Baseline
TEWL (g/h/m^2^) ^a^	Baseline	18.9 ± 5.4	−1.6 ± 4.6	19.2 ± 4.7	−2.8± 3.5
	6 weeks	17.3 ± 5.3		16.4 ± 4.5 ^#^	
Hydration ^a^	Baseline	38.1 ± 17.8	−0.6 ± 14.7	36.7 ± 11.8	0.8 ± 14.9
	6 weeks	37.5 ± 14.8		37.4 ± 17.0	
pH ^b^	Baseline	6.0 ± 0.2	0.01 ± 0.43	6.0 ± 0.2	0.1 ± 0.3
	6 weeks	6.0 ± 0.3		6.1 ± 0.3	
Melanin Index	Baseline	151.1 ± 41.9	−7.9 ± 29.7	156.9 ± 46.0	−7.3 ± 30.9
	6 weeks	143.2 ± 37.9		149.6 ± 46.3	
Erythema Index ^b^	Baseline	398.7 ± 112.8	−3.2 ± 67.3	415.3 ± 108.2	−25.3 ± 62.3
	6 weeks	395.5 ± 99.0		390.0 ± 120.0	
L*	Baseline	59.8 ± 4.5	−0.8 ± 3.1	59.2 ± 3.4	-0.6 ± 2.6
	6 weeks	59.0 ± 3.7		58.7 ± 4.3	
a*	Baseline	15.1 ± 3.1	0.3 ± 1.5	14.9 ± 1.8	0.6 ± 2.2
	6 weeks	15.4 ± 2.9		15.6 ± 2.9	
b* ^a^	Baseline	13.9 ± 2.5	−0.2 ± 2.1	13.6 ± 2.7	−0.1 ± 2.1
	6 weeks	13.6 ± 2.6		13.4 ± 2.7	
Gross Elasticity (%)	Baseline	70.2 ± 9.7	−0.1 ± 11.6	68.7 ± 10.5	4.1 ± 9.9
	6 weeks	70.0 ± 12.1		72.8 ± 9.6	
Net Elasticity ^a^ (%)	Baseline	50.5 ± 11.6	−0.2 ± 13.3	47.7 ± 10.8	8.1 ± 12.3 ^&^
	6 weeks	50.3 ± 13.0		55.7 ± 11.6 ^#,^^	
Viscoelasticity	Baseline	29.3 ± 7.5	−2.7 ± 8.5	29.5 ± 5.3	−2.7 ± 6.7
	6 weeks	26.6 ± 6.5		26.8 ± 4.1	
Biological Elasticity (%)	Baseline	38.1 ± 8.5	2.4 ± 8.9	36.7 ± 8.7	5.8 ± 10.7
	6 weeks	40.5 ± 10.3		42.5 ± 11.1 ^#^	
Wrinkle ^a^	Baseline	115.2 ± 25.0	−4.6 ± 20.4	116.3 ± 27.9	−7.6 ± 12.9
	6 weeks	110.7 ± 20.8		108.7 ± 26.8	
Smoothness	Baseline	292.5 ± 89.6	−27.0 ± 60.6	267.8 ± 50.1 ^	5.5 ± 33.8 ^&^
	6 weeks	265.4 ± 49.0 ^#^		273.3 ± 59.8	
Roughness ^a^	Baseline	2.86 ± 0.64	0.44 ± 1.02	3.00 ± 0.94	0.03 ± 0.83
	6 weeks	3.30 ± 1.07		3.03 ± 0.97	
Scaliness	Baseline	0.22 ± 0.44	−0.09 ± 0.39	0.18 ± 0.32	−0.04 ± 0.22
	6 weeks	0.14 ± 0.19		0.13 ± 0.19	

Data are expressed as means ± SD (*n* = 22) and organized by time and treatment. ^a^ Log transformed for statistical comparison; ^b^ Carryover effect was accounted in mixed effect model; ^#^ Significant difference between baseline and after six weeks in the same treatment groups by post hoc contrast; ^ is the significant difference between cranberry beverage and placebo at the same time point by post hoc contrast; ^&^ is the significant difference between change from baseline to final cranberry beverage and placebo by post hoc linear contrast.

**Table 3:** Skin parameters on the forearm in women who consumed cranberry beverage or placebo for six weeks.

Skin Parameters	Time Period	Placebo	Change from Baseline	Cranberry Beverage	Change from Baseline
TEWL (g/h/m^2^) ^a^	Baseline	7.6 ± 3.5	0.4 ± 4.1	7.8 ± 2.8	−1.3 ± 2.3
	6 weeks	8.1 ± 5.7		6.5 ± 1.6	
Hydration	Baseline	39.1 ± 10.4	2.8 ± 10.8	38.7 ± 7.5	1.7 ± 7.2
	6 weeks	41.9 ± 9.7		40.4 ± 8.1	
pH	Baseline	5.55 ± 0.54	0.004 ± 0.6	5.57 ± 0.45	0.1 ± 0.5
	6 weeks	5.55 ± 0.38		5.69 ± 0.51	
Melanin Index ^a^	Baseline	124.8 ± 39.3	−1.9 ± 15.0	131.9 ± 42.5	−4.6 ± 32.9
	6 weeks	122.9 ± 48.8		127.3 ± 44.4	
Erythema Index	Baseline	199.0 ± 48.7	−3.0 ± 43.6	203.5 ± 56.0	4.9 ± 33.1
	6 weeks	196.0 ± 69.5		208.4 ± 65.5	
L* ^a^	Baseline	65.2 ± 4.1	0.1 ± 1.7	65.2 ± 4.0	−0.5 ± 1.8
	6 weeks	65.3 ± 4.5		64.7 ± 4.6	
a*	Baseline	7.99 ± 1.33	0.1 ± 0.8	8.18 ± 1.37	0.3 ± 0.7
	6 weeks	8.13 ± 1.32		8.44 ± 1.33	
b*	Baseline	13.9 ± 2.4	0.3 ± 1.2	13.7 ± 3.4	0.3 ± 1.1
	6 weeks	14.2 ± 3.1		14.0 ± 3.5	
Gross Elasticity ^a,b^ (%)	Baseline	81.4 ± 9.3	−0.6 ± 6.8	78.2 ± 9.9 ^	5.4 ± 6.1 ^&^
	6 weeks	80.8 ± 11.6		83.5 ± 7.2 ^#,^^	
Net Elasticity (%)	Baseline	78.2 ± 12.7	0.1 ± 7.5	76.1 ± 12.2	7.0 ± 9.1 ^&^
	6 weeks	78.3 ± 12.4		83.1 ± 9.0 ^#,^^	
Viscoelasticity ^a,b^	Baseline	29.9 ± 5.4	0.2 ± 6.6	34.4 ± 14.6	−5.9 ± 14.1
	6 weeks	30.2 ± 7.56		28.5 ± 7.2 ^#^	
Biological Elasticity ^b^ (%)	Baseline	60.3 ± 10.2	0.4 ± 7.2	58.6 ± 12.4	4.5 ± 7.5
	6 weeks	60.7 ± 11.2		63.1 ± 9.3 ^#^	
Wrinkle	Baseline	71.6 ± 16.2	−2.9 ± 16.6	71.0 ± 19.9	−2.6 ± 9.8
	6 weeks	68.7 ± 22.3		68.4 ± 18.3	
Smoothness ^a^	Baseline	218.0 ± 37.7	−6.2 ± 48.6	229.3 ± 43.9	−16.1 ± 45.5
	6 weeks	211.8 ± 55.7		213.2 ± 48.8	
Roughness	Baseline	1.58 ± 0.64	−0.04 ± 0.44	1.82 ± 1.14	−0.15 ± 1.27
	6 weeks	1.55 ± 0.70		1.66 ± 0.81	
Scaliness ^a^	Baseline	0.17 ± 0.20	0.02 ± 0.18	0.18 ± 0.25	−0.04 ± 0.11
	6 weeks	0.18 ± 0.22		0.14 ± 0.24	

Data are expressed as means± SD (*n* = 22) and organized by time and treatment. ^a^ Log transformed before statistical comparison for statistical comparison; ^b^ carryover effect was accounted in mixed effect model; ^#^ is the significant difference between baseline and after six weeks in the same treatment groups by post hoc contrast; ^ is the significant difference between cranberry beverage and placebo at the same time point by post hoc contrast; ^&^ is the significant difference between change from baseline to final cranberry beverage and placebo by post hoc linear contrast.

**Table 4:** Discriminant lipids after cranberry beverage and placebo consumption based on paired PLS-DA models (VIP ≥ 1.50) combined with univariate analysis (*p*-value ≤ 0.05 by paired *t*-test).

		Cranberry Beverage Baseline vs. Final			Placebo Baseline vs. Final			Cranberry Beverage Final vs. Placebo Final		
Class ^a^	Lipids	VIP ^b^	Change ^c^	*p*-Value ^d^	VIP ^b^	Change ^c^	*p*-Value ^d^	VIP ^b^	Change ^c^	*p*-Value ^d^
FA	FA18:2_SC	0.58	-	0.13	1.65	↑	<0.01	0.37	-	0.23
FA	FA20:0_SC	1.57	↑	<0.01	2.08	↑	<0.01	0.48	-	0.59
NDS	DS(C18)18:0	0.92	-	0.66	1.69	↑	<0.01	1.76	-	0.22
NDS	DS(C19)26:0	0.33	-	0.40	0.18	-	0.73	1.57	↑	0.04
NDS	DS(C20)23:0	2.01	↑	<0.01	1.16	-	0.02	0.92	-	0.26
NDS	DS(C20)25:0	1.78	↑	<0.01	1.91	↑	<0.01	0.96	-	0.82
NDS	DS(C23)26:0	1.61	↑	<0.01	1.27	-	0.59	0.96	-	0.25
NDS	DS(C24)24:0	1.72	↑	<0.01	1.2	-	0.56	0.95	-	0.40
NDS	DS(C26)24:0	1.61	↑	<0.01	0.91	-	0.36	1.11	-	0.15
AH	H(C21)a25:0	1.51	↓	0.03	0.26	-	0.67	1.83	-	0.14
EOH	H(C18)w30:0_18:2	1.52	↑	<0.01	0.54	-	0.31	0.47	-	0.56
EOH	H(C23)w30:0_18:2	1.86	↑	<0.01	0.26	-	0.67	1.09	-	0.17
EOH	H(C23)w32:0_18:2	1.79	↑	<0.01	1.27	-	0.02	0.44	-	0.62
EOH	H(C23)w34:0_18:2	1.67	↑	0.01	0.75	-	0.57	0.99	-	0.38
NP	P(C18)29:0	0.45	-	0.95	1.34	-	0.02	1.80	↓	0.04
NS	S(C19)28:0	1.11	-	0.07	1.91	↑	<0.01	1.86	-	0.07
NS	S(C21)25:0	1.52	↑	<0.01	0.94	-	0.13	1.02	-	0.22
EOS	S(C21)w32:0_18:2	1.01	-	0.39	1.55	-	0.13	1.65	↑	0.03
EOS	S(C21)w34:0_18:2	1.52	-	0.13	2.03	↓	<0.01	0.83	-	0.32
EOS	S(C22)w30:0_18:2	0.31	-	0.90	2.19	↓	0.01	1.99	↑	0.01

^a^ Class of stratum corneum lipids (FA: Fatty acid, NDS: Non-hydroxy-dihydrosphingosine, ADS: α-hydroxy-dihydrosphingosine, AH: α-hydroxy-6-hydroxy-sphingosine, EOH: ester-linked-omega-hydroxy-6-hydroxy-sphingosine, NP: non-hydroxy-phytoceramide, AP: α-hydroxy-phytoceramide, NS: non-hydroxy-sphingosine, AS: α-hydroxy-sphingosine, EOS: ester-linked-omega-hydroxy-sphingosine); ^b^ VIP score calculated by PLS-DA; ^c^ change only shown in VIP > 1.5 was determined based on cranberry beverage final vs. baseline, placebo final vs. baseline, and cranberry beverage vs. placebo final; ^d^ *p*-value was calculated from paired *t*-test. ↑ Indicates an increase, ↓ indicates a decrease.

## Data Availability

The data contained within this article will be made available upon reasonable request.

## Supplementary Materials

The following supporting information can be downloaded at: https://www.mdpi.com/article/10.3390/nu16183126/s1, Figure S1: (A) Experimental flow chart and (B) sample collection timeline. Sample collection included blood, skin-stripping for lipids, and skin swabs for microbiomes. Figure S2: Visioscan images from a participant (≥40 years old) at baselines and final time points after six weeks of cranberry beverage (A,B) and placebo (C,D). Figure S3: Plasma levels of SOD (A), GPx (B), AGE (C), TNF-α (D), IL-17 (E), and Hs-CRP (F) in women stratified by age (*n* = 11 for <40 years old and *n* = 11 for ≥40 years old). Plasma was collected at baseline and final time points after six weeks of cranberry beverage or placebo consumption. Results are expressed as mean ± SD. & is the significant difference between cranberry beverage and placebo; # is the significant difference between final and baseline. SOD: superoxide dismutase, GPx: glutathione peroxide; IL-interleukin; TNF-α: tumor necrosis factor-α; Hs-CRP: high-sensitivity C-reactive protein. Figure S4: PCA score plots of skin lipids in all four groups. t[1] represents the first principal component; t[2] represents the second component. Figure S5: Validation plots of paired PLS-DA models for Figure 3 obtained from 200 permutation tests. The X-axis is the correlation coefficient between the permutated and original response variables. The Y-axis represents the value of R2 (variance of Y explained by the model) and Q2 (the model’s predictive ability). (A) differences between cranberry beverage baseline and final; (C) differences between cranberry beverage final and placebo final; (B) differences between placebo baseline and final. Figure S6: Cranberry beverage intake did not affect α- or β-diversity compared to baseline or placebo. α-diversity was measured by Chao1, Shannon, and Simpson Index (A). β-diversity was assessed using principal coordinate analysis of Bray–Curtis dissimilarity index (B) and Jaccard similarity index (C). Figure S7: Relative abundance (RA%) of the top bacteria at the A) phylum, B) family, C) genus level.; Table S1: Phenolic, organic acid, and sugar content in cranberry beverage and placebo [50,51,52,53]. Table S2: Baseline characteristics of the participants. Table S3: Dietary intake measured by food frequency questionnaires among participants at baseline and after six weeks of drinking cranberry beverages or placebo. Table S4: Change in redness of the skin (∆a*) caused by UVB irradiation (2× MED) before and after 6 weeks of consumption of cranberry beverage or placebo in participants stratified by age. Table S5: Skin parameters measured on the face in women who consumed cranberry beverage or placebo for six weeks in participants stratified by age. Table S6: Skin parameters measured on the forearm in women who consumed cranberry beverage or placebo for six weeks in participants stratified by age. Table S7: Summary of parameters for partial least squares discriminant (PLS-DA) models describing the overall differences in skin lipidomics affected by cranberry beverage and placebo.

## Abbreviations

ADS	α-hydroxy-dihydrosphingosine
AGE	Advanced glycation products
AH	α-hydroxy-6-hydroxy-sphingosine
AP	α-hydroxy-phytoceramide
AS	α-hydroxy-sphingosine
BMI	Body mass index
ELISA	Enzyme-linked immunosorbent assay
EOH	Ester-linked-omega-hydroxy-6-hydroxy-sphingosine
EOS	Ester-linked-omega-hydroxy-sphingosine
GPx	Glutathione peroxide
Hs-CRP	High-sensitivity C-reactive protein
IL	Interleukin
MED	Minimal erythema dose
NP	Non-hydroxy-phytoceramide
NS	Non-hydroxy-sphingosine
PCA	Principal component analysis
PLS-DA	Partial least squares discriminant analysis
ROSs	Reactive oxygen species
SC	Stratum corneum
SOD	superoxide dismutase
TEWL	Transepidermal water loss
TNF-α	Tumor necrosis factor-α
UVB	Ultraviolet B
