# Supplementary material for: Polyphenol-Rich Cranberry Beverage Positively Affected Skin Health, Skin Lipids, Skin Microbiome, Inflammation, and Oxidative Stress in Women in a Randomized Controlled Trial
Source: Nutrients. 2024 Sep 16;16(18):3126. doi: 10.3390/nu16183126 (PMC11434900; doi:10.3390/nu16183126)
Supplement: Supplementary file 1 [file nutrients-16-03126-s001.zip › Supplemental Figures Gu edit 091524.pdf]

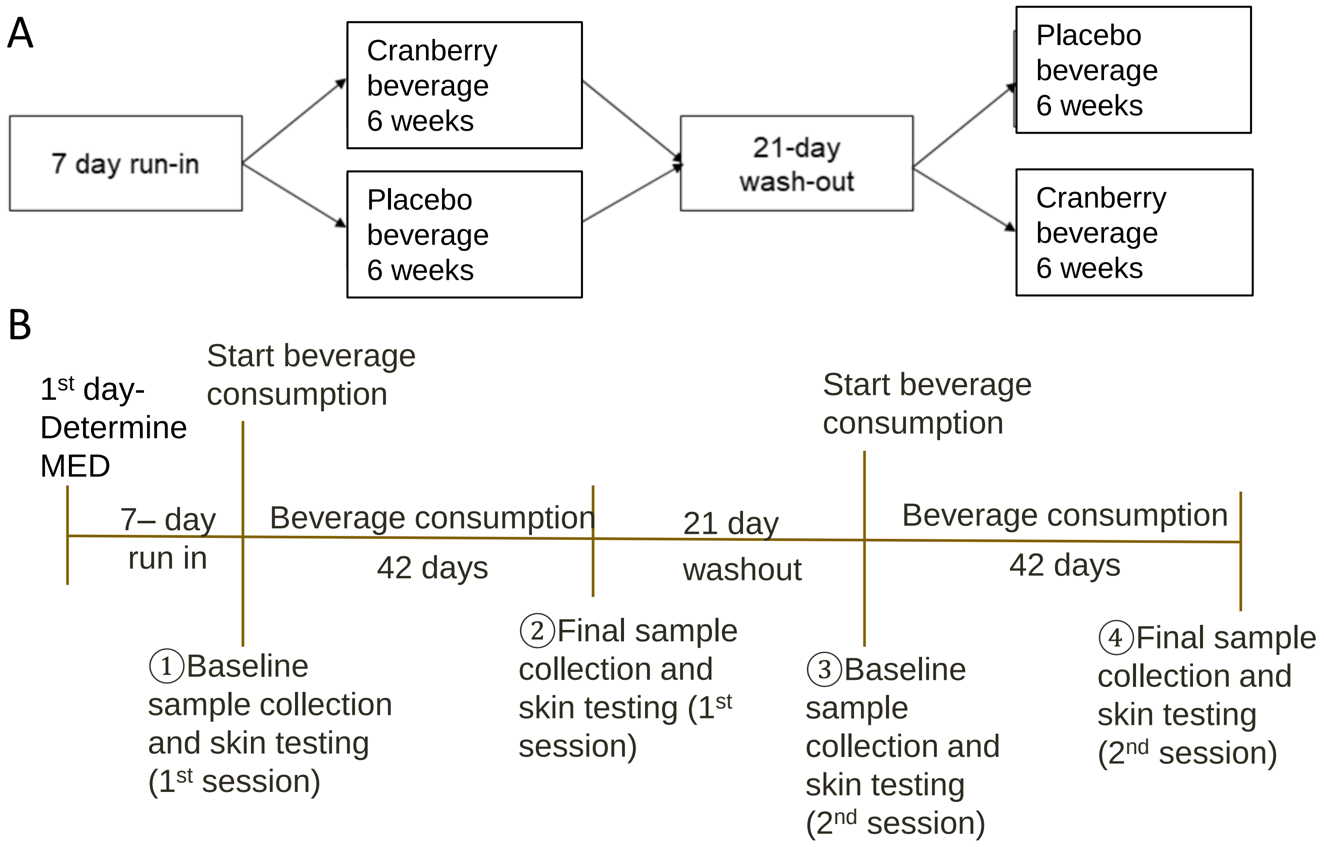

Figure S1. A) Experimental flow chart and B) sample collection timeline. Sample collection included blood, skin-stripping for lipids, and skin swabs for microbiomes. MED:minimal erythema dose.

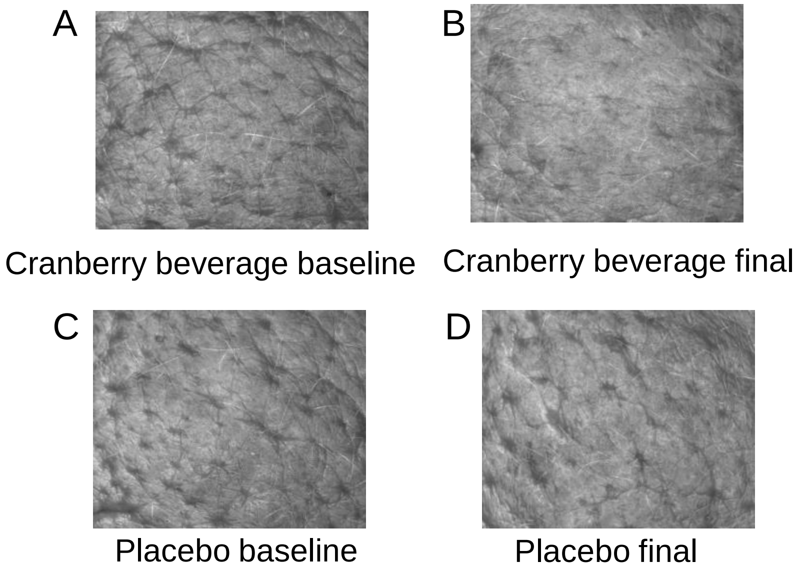

Figure S2. Visioscan images from a participant (≥40 years old) at baselines and final time points after six weeks of cranberry beverage (A, B) and placebo (C, D).

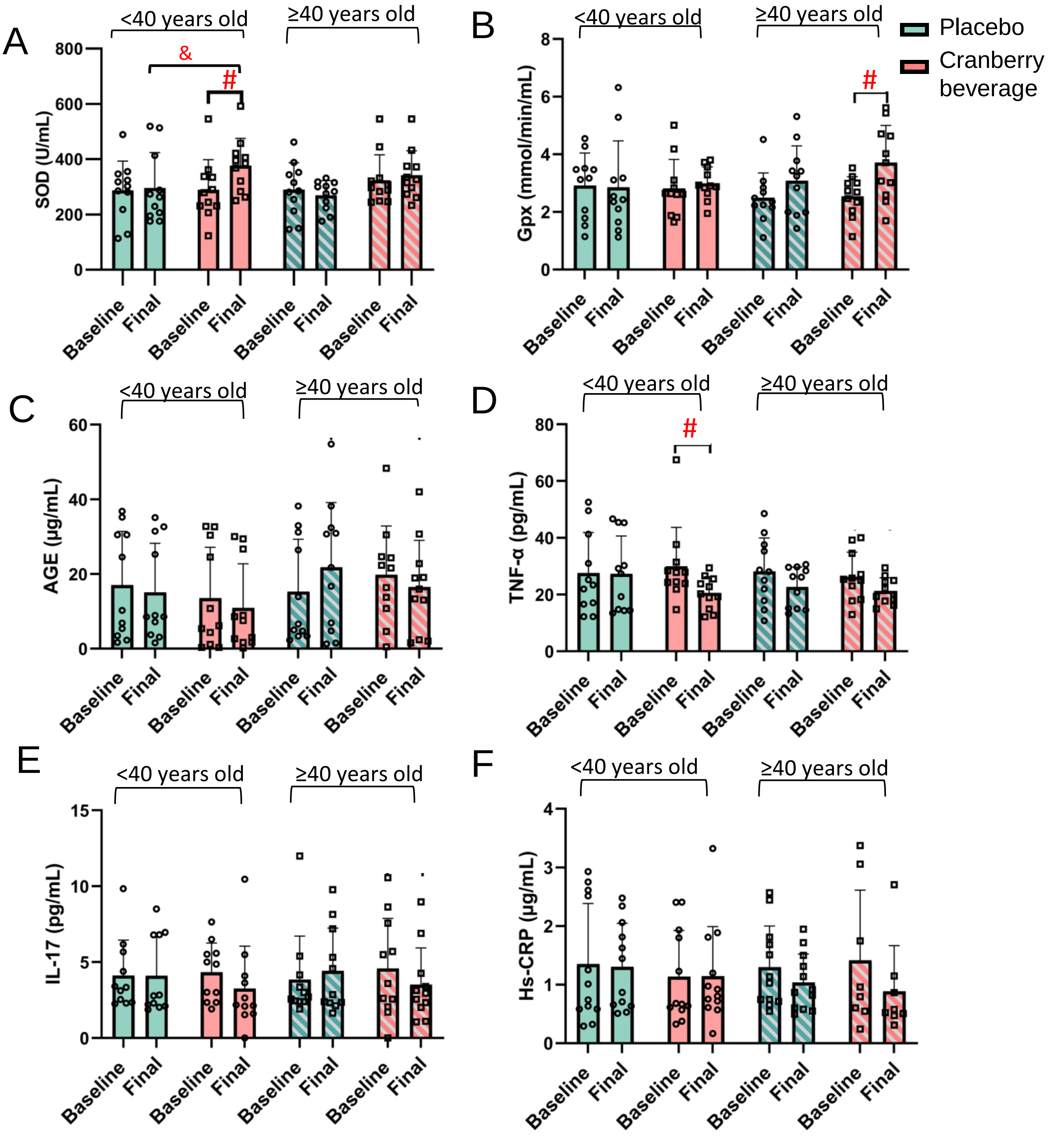

Figure S3. Plasma levels of SOD (A), GPx (B), AGE (C), TNF-α (D), IL-17 (E), and Hs-CRP (F) in women stratified by age (n=11 for <40 years old and n=11 for ≥40 years old). Plasma was collected at baseline and final time points after six weeks of cranberry beverage or placebo consumption. Results are expressed as mean±SD. & Significant difference between cranberry beverage and placebo; # Significant difference between final and baseline; SOD: superoxide dismutase, GPx: glutathione peroxide; IL-interleukin; TNF-α: tumor necrosis factor-α; Hs-CRP: high sensitivity C-reactive protein.

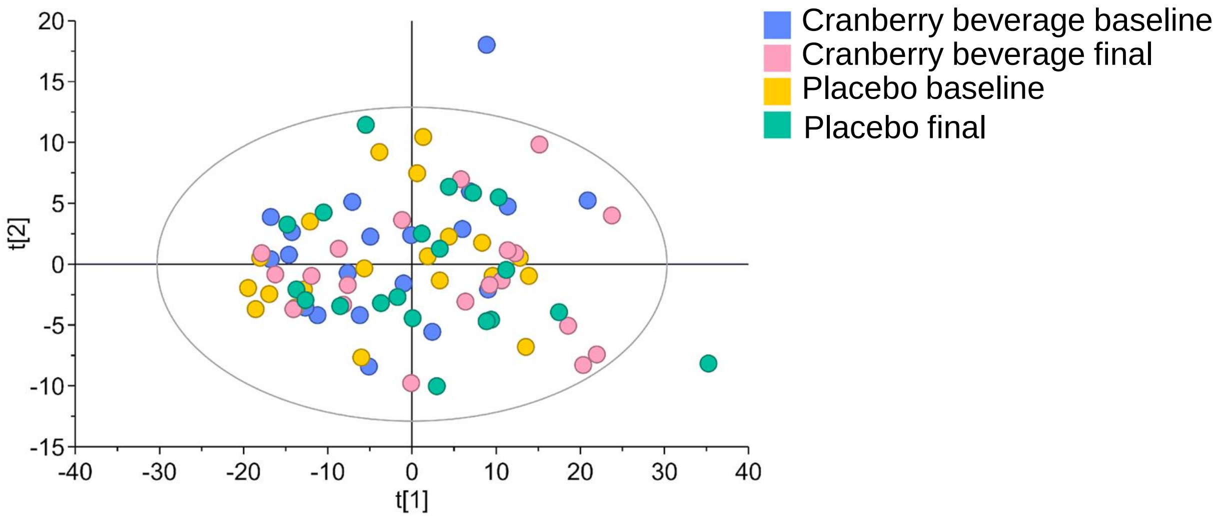

Figure S4. PCA score plot of skin lipids in all four groups. t[1] represents the first principal component; t[2] represents the second component.

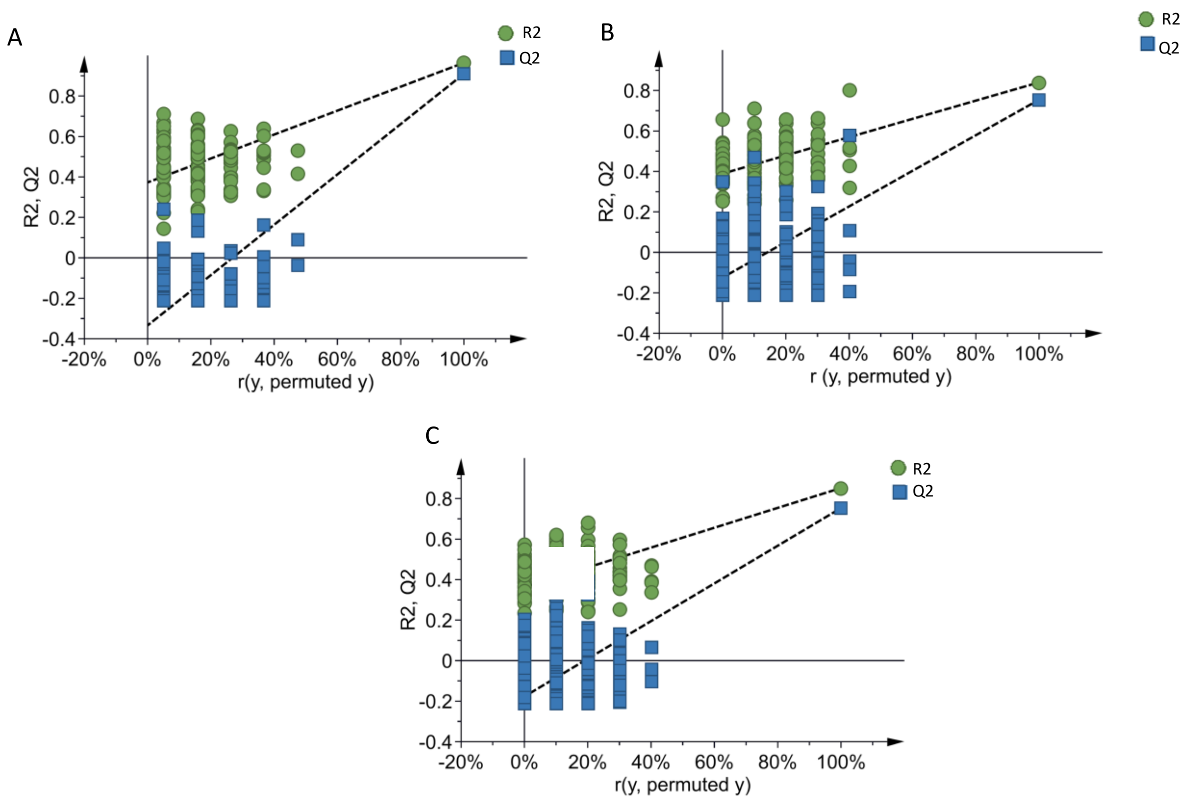

Figure S5. Validation plots of paired PLS-DA models for **Figure 3** obtained from 200 permutation tests. The X-axis is the correlation coefficient between the permutated and original response variables. The Y-axis represents the value of R2 (variance of Y explained by the model) and Q2 (the model's predictive ability). (A) differences between cranberry beverage baseline and final; (C) differences between cranberry beverage final and placebo final; (B) differences between placebo baseline and final.

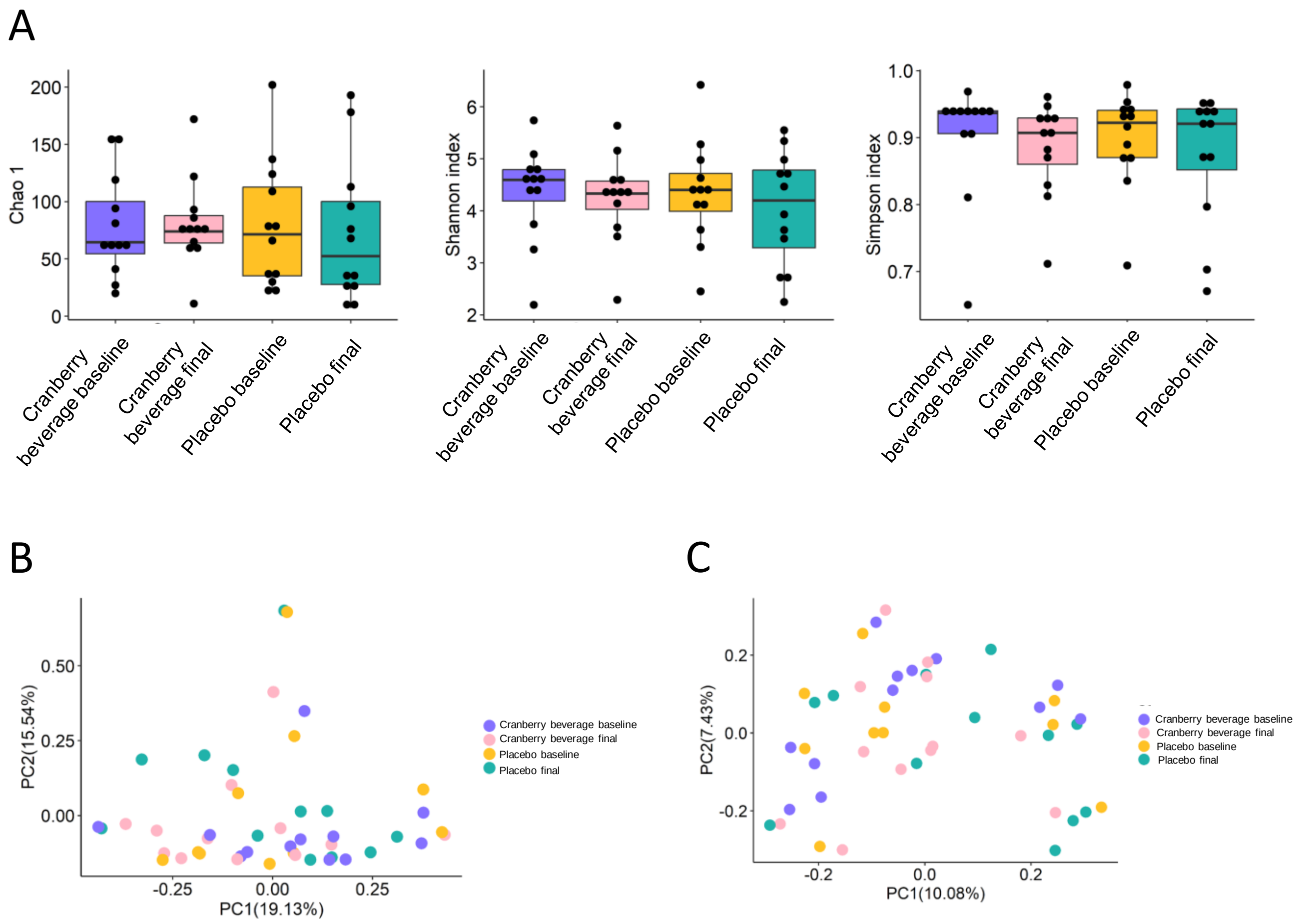

Figure S6. Cranberry beverage intake did not affect α- or β-diversity compared to baseline or placebo. α-diversity was measured by Chao1, Shannon, and Simpson Index (A). β-diversity was assessed using principal coordinate analysis of Bray-Curtis dissimilarity index (B) and Jaccard similarity index (C).

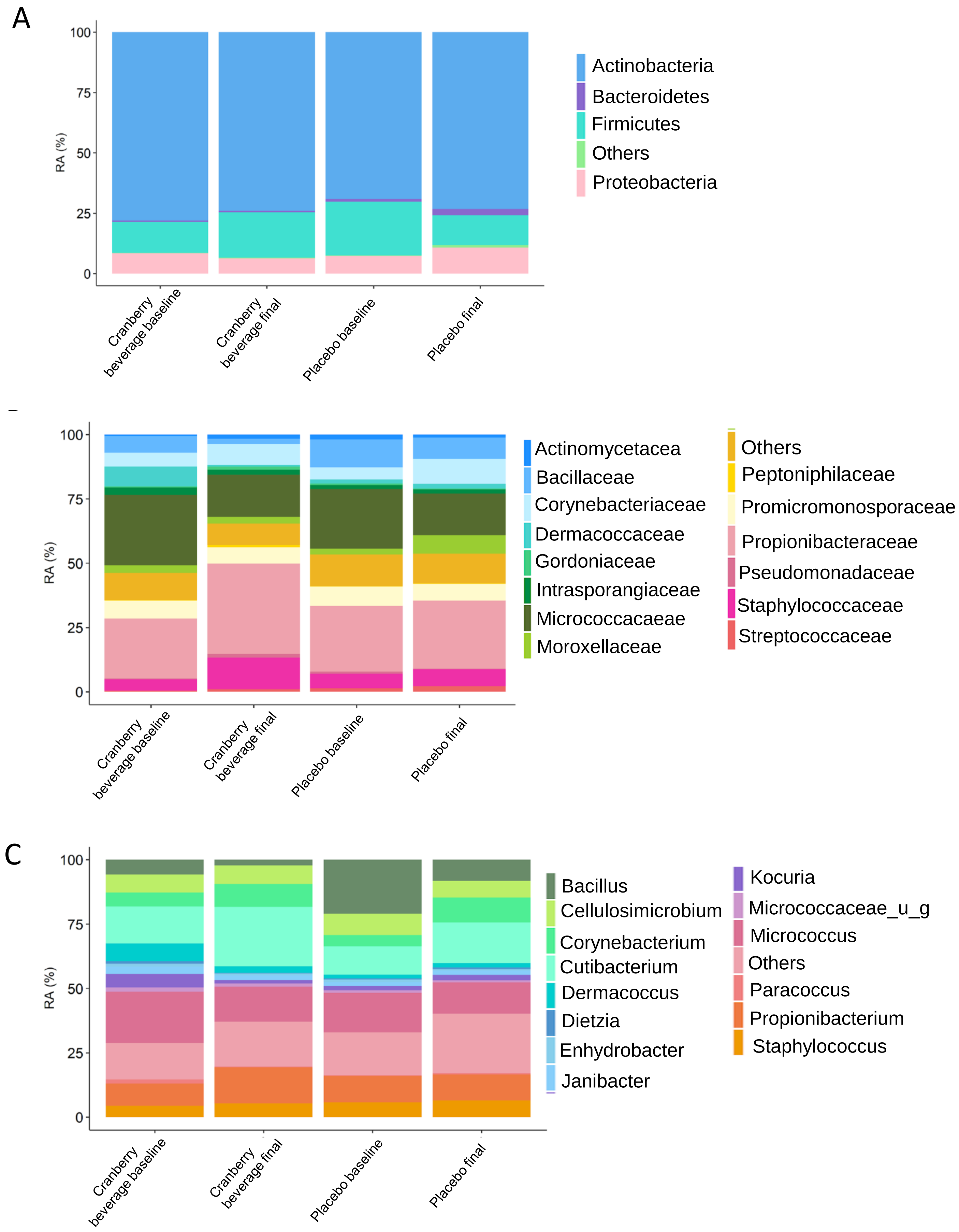

Figure S7. Relative abundance (RA%) of the major skin bacteria at the A) phylum, B) family, C) genus level.
