# Supplementary material for: Polyphenol-Rich Cranberry Beverage Positively Affected Skin Health, Skin Lipids, Skin Microbiome, Inflammation, and Oxidative Stress in Women in a Randomized Controlled Trial
Source: Nutrients. 2024 Sep 16;16(18):3126. doi: 10.3390/nu16183126 (PMC11434900; doi:10.3390/nu16183126)
Supplement: Supplementary file 1 [file nutrients-16-03126-s001.zip › Supplementary Methods and Tables 091524.pdf]

Supplementary methods and tables (S1-S7)

Skin lipid sampling and analysis

More than skin 350350 skin lipids, including ceramides, free fatty acids, cholesterol, and cholesterol sulfate, were found using TrueMass Stratum Corneum Lipid Panel from Metabolon (Waters Corporation, Milford, MA/ Sciex, Framingham, MA; Metabolon, Inc, Durham, NC).

Squame discs containing skin lipids were extracted in three batches with an organic solvent after adding a known amount of surrogate standard. The extracts were then combined and evaporated to dryness. The dried extract was then reconstituted and analyzed using a Waters UPC^2/Sciex QTrap 5500 mass spectrometer SFC-MS/MS system in MRM mode using characteristic parent fragment mass transitions for each analyte trace.

Lipids were quantified based on peak area comparisons of the individual lipid species with their corresponding surrogate standards. Ceramide measures were semi-quantitative due to the lack of a full set of standards to cover all ceramide classes and chain lengths. Data quality was assessed by precision evaluation based on the median % coefficient of variance of the quality control sample. The observed median % coefficient of variance was 17.6%, which is below their targeted acceptance criteria of quality control precision of 25.0%.

The lipid classes identified in this analysis included the ceramides, α-hydroxy-dihydrosphingosine (ADS), α-hydroxy-6-hydroxy-sphingosine (AH), α-hydroxy-phytoceramide (AP), α-hydroxy-sphingosine (AS), ester-linked-omega-hydroxy-6-hydroxy-sphingosine (EOH), ester-linked-omega-hydroxy-sphingosin (EOS), non-

hydroxy-dihydrosphingosine (NDS), non-hydroxy-phytoceramide (NP), Non-hydroxy-sphingosine (NS), cholesterol, and cholesterol sulfate. Ceramides names are based on their sphingoid backbone and associated fatty acid species. For example, in the EOH class, H(C18)w30:0_18:2, indicates a 6-hydroxysphinosine backbone with a saturated C30 omega-hydroxy fatty acid esterifed with an unsaturated C18 fatty acid with two double bonds.

Cutaneous microbiota sampling and analysis

Samples were pre-treated with metapolyzyme and extracted with Qiagen DNAeasy Powersoil Pro according to the manufacturer's protocol (Qiagen, Venlo, Netherlands). Multiplexing was done using unique dual index barcodes and the Illumina universal adapter sequence. DNA was prepared and sequenced using Illumina Hiseq 200 platform.

The relative abundance of each taxon was analyzed using the MaAsLin2 R package. Relative abundance data was log-transformed, and analysis in MaAsLin2 was performed using default parameters (taxonomic feature prevalent in a minimum of 10% of samples, the minimum relative abundance of 0.01%, $p \leq 0.05$, $q \leq 0.25$) with group as fixed effects and participant as a random effect.

MaAsLin2 was also used to find significant multivariate associations between significantly changed microbial taxa and skin parameters.

Correlations were analyzed using Spearman correlation. This was implemented using the cor function in R. Significance was set at $p \leq 0.05$.

Supplemental tables (S1-S7)

Table S1. Phenolic, organic acid, and sugar content in cranberry beverage and placebo.

Compound class	Compounds	Cranberry beverage	Placebo
Anthocyanins (µg/mL) [a]	Cyanidin-3-galactoside	24.18± 0.19	nd
	Cyanidin-3-glucoside	nd	nd
	Cyanidin-3-arabinoside	13.92±0.20	nd
	Peonidin-3-galactoside	28.76±0.25	nd
	Peonidin-3-glucoside	2.39±0.02	nd
	Peonidin-3-arabinoside	13.07±0.21	nd
Total procyanidins (mg/8 oz)	OSC-DMAC [b]	192.88±1.45	nd
	BL-DMAC [c]	64.22±3.04	nd
Total phenols (mg/8 oz) [d]		171.94±1.77	50.79±0.18
Phenolics (µg/mL) [a]	(-)-Epicatechin	nd	nd
	2-hydroxybenzoic acid	nd	nd
	3,4-dihyrdoxybenzoic Acid	nd	nd
	4-hydroxycinnamic Acid	nd	nd
	Benzoic Acid	10.94±0.06	nd
	Caffeic Acid	nd	nd
	(+)-Catechin	nd	nd
	Chlorogenic Acid	6.29±0.01	nd
	Ellagic Acid	nd	nd
	Ferulic Acid	nd	nd
	Gallic Acid	nd	nd
	Myricetin	nd	nd
	Myricetin 3-galactoside	22.06±0.09	nd
	Myricetin 3-rhamnoside	5.86±0.31	nd
	Quercetin	8.07±0.01	nd
	Quercetin-3-galactoside	53.44±0.23	nd
	Quercetin-3-rhamnoside	12.76±0.03	nd
	Cinnamic Acid	nd	nd
	Vanillic Acid	nd	nd
Organic Acid (g/8 oz) [a]	Citric Acid	0.14±0.00	0.69±0.02
	Malic Acid	0.10±0.00	nd
	Quinic Acid	0.14±0.00	nd
	Fumaric Acid	nd	nd
	Oxalic Acid	nd	nd
	Isocitric acid	nd	nd
	Total	0.38	0.69
Sugar (g/8 oz) [a]	Glycerin	<0.71	<0.71
	Sucrose	<0.24	<0.24
	Fructose	1.28±0.13	1.17±0.08
	Dextrose	nd	nd
	Sorbitol	<0.24	<0.24

Values are means± standard deviation for the duplicate measures provided by Ocean Spray Inc. nd, not detected. [a] Anthocyanins, phenolics, organic acids, and sugars were analyzed by high-performance liquid chromatography [1,2]. [b] measured with a colorimetric assay (OSC-DMAC) utilizing an isolated fraction of purified cranberry procyanidins as a reference standard [3]. [c] measuring with a colorimetric assay (BL-DMAC) utilizing procyanidin A2 dimer as a reference standard [4]. [d] Total Phenolics measured using a Folin-Ciocalteu method.

Table S2. Baseline characteristics of the participants.

	Placebo-Cranberry beverage	Cranberry beverage-placebo	p-values
Age (yr)	39.0±12.7	37.8±12.2	0.85
Weight (kg)	73.8±8.6	67.6±9.2	0.12
BMI (kg/m^2)	25.9±2.8	25.1±2.4	0.47
Fitzpatrick skin type	2.7±0.5	2.6±0.5	0.67
MED (J/cm^2)	0.5±0.3	0.6±0.5	0.50
Number of participants	11	11	

Participants in the placebo-cranberry beverage group were given a placebo during the first phase and then switched to cranberry beverage for the second session. The opposite sequence was used for the cranberry beverage-placebo group. Data are mean± SD. BMI: body mass index; MED: minimal erythema dose.

Table S3. Dietary intake measured by food frequency questionnaires among participants at baseline and after six weeks of drinking cranberry beverage or placebo.

	Frequency	Time Period	Placebo	Cranberry beverage
Bread	Serving/day	Baseline	0.5±0.5	0.5±0.6
		6 weeks	0.4±0.7	0.4±0.7
Vegetables	Serving/day	Baseline	1.5±0.6	1.5±0.7
		6 weeks	1.5±0.7	1.5±0.6
Fruit	Serving/day	Baseline	1.2±0.7	1.2±0.8
		6 weeks	1.2 ±0.8	1.1±0.8
Milk or Yogurt	Serving/day	Baseline	0.6±0.6	0.7±0.7
		6 weeks	0.7±0.5	0.7±0.8
Rice or Pasta	Serving/day	Baseline	0.5±0.7	0.4±0.5
		6 weeks	0.5±0.7	0.6±0.7
Vegetable oils	Serving/day	Baseline	0.3±0.5	0.5±0.7
		6 weeks	0.3±0.5	0.4±0.7
Alcoholic beverages	Serving/day	Baseline	0.1±0.3	0.1±0.3
		6 weeks	0.1±0.3	0.1±0.3
Breakfast cereals	Serving/day	Baseline	0.1±0.3	0.1±0.3
		6 weeks	0.2±0.4	0.1±0.3
Meat	Serving/week	Baseline	4.0±2.0	3.8±1.9
		6 weeks	4.0±2.0	4.0±2.0
Sausages	Serving/week	Baseline	2.4±1.2	2.3±0.9
		6 weeks	2.4±1.2	2.3±0.9
Cheese	Serving/week	Baseline	3.7±1.7	3.9±1.9
		6 weeks	3.7±1.7	4.1±2.0
Animal fat (butter, lard)	Serving/week	Baseline	2.9±1.7	3.2±1.7
		6 weeks	2.9±1.7	3.3±1.7
Vegetable oil	Serving/week	Baseline	3.2±1.9	3.1±1.7
		6 weeks	3.2±1.9	3.0±1.6
Fish	Serving/week	Baseline	1.3±0.6	1.3±0.6
		6 weeks	1.2±0.5	1.4±0.6
Legumes	Serving/week	Baseline	2.0±1.0	2.0±0.9
		6 weeks	1.9±1.10	2.3±1.2
Nuts	Serving/week	Baseline	1.9±1.2	1.9±1.2
		6 weeks	2.0±1.2	2.2±1.2

Results are based on a validated self-reported semi-quantitative food frequency questionnaire. Participants filled out the questionnaire before and after each six-week session to determine whether there were overall changes in diet throughout the trial. Data is expressed as mean± SD (n=22) and organized by time and treatment.

Table S4. Change in redness of the skin (Δa*) caused by UVB irradiation (2× MED) before and after 6 weeks of consumption of cranberry beverage or placebo in participants stratified by age.

		<40 years old (n=11)				≥40 years old (n=11)			
	Time Period	Placebo	Placebo change from baseline	Cranberry beverage	Cranberry change from baseline	Placebo	Placebo change from baseline	Cranberry beverage	Cranberry change from baseline
Δa*	Baseline	8.5± 2.6	-0.7±1.2	7.7±2.9	-0.8±2.9	9.1±3.4	0.7±4.3	10.9±2.6	-3.4±2.2&
	6 weeks	7.8± 3.0		6.9±4.4		9.9±2.8		7.5±2.4#,^	

Data are expressed as means± standard deviation (n=11) and organized by time and treatment. Change from baseline = $\Delta a^*_{6\ week}$ - $\Delta a^*_{Baseline}$. ^ Significant difference between cranberry beverage and placebo at the same time point by post hoc contrast.

[#]Significant difference between cranberry beverage and placebo baseline after six weeks by post hoc contrast.

[&]Significant difference between cranberry beverage and placebo by post-hoc linear contrast.

Table S5. Skin parameters on the face in women who consumed cranberry beverage or placebo for six weeks in participants stratified by age.

Skin parameters	Time Period	<40 years old (n=11)				≥40 years old (n=11)			
		Placebo	Change from baseline	Cranberry beverage	Change from baseline	Placebo	Change from baseline	Cranberry beverage	Change from baseline
TEWL (g/h/m^2)[a]	Baseline	15.6±3.2	-0.9± 1.4	16.7±4.4	-2.0± 1.5	22.2±5.2	-2.3±6.5	21.7±3.4	-3.5±4.7
	6 weeks	14.7±3.0		14.6±4.1		19.8±5.9		18.3±4.4#	
Hydration [a]	Baseline	40.0±19.3	2.4±15.7	37.4±12.6	3.7± 16.5	36.2±17.0	-3.6±13.7	35.9±11.6	-2.1±13.5
	6 weeks	42.4±16.4		41.1±16.8		32.6±11.6		33.8±16.3	
pH	Baseline	5.97±0.21	0.04±0.31	6.01±0.27	0.16±0.30	6.01±0.23	-0.01 ±0.54	5.96±0.21	0.07±0.26
	6 weeks	6.00±0.23		6.16±0.36		6.01±0.42		6.03±0.26	
Melanin Index[a]	Baseline	141.1±35.1	-18.0 ±26.8	130.9±36.6	-4.5±34.5	161.1±47.4	2.3±30.1	182.9±40.3	-10.2± 28.5
	6 weeks	123.1±16.3		126.4±21.6		163.3±43.3		172.8±53.4	
Erythema Index	Baseline	376.2±123.3	-1.1±51.5	391.2±123.4	-38.5± 82.6	421.3±102.3	-5.3±82.7	439.4±89.9	-12.1± 30.6
	6 weeks	375.1±128.7		352.7±141.7		416.0±56.0		427.3±84.1	
L*	Baseline	61.3±4.4	-0.9±2.4	61.2±2.9	-0.6±3.6	58.3±4.1	-0.7±3.7	57.3±2.8	-0.5±1.1
	6 weeks	60.4±4.3		60.6±4.4		57.6±2.4		56.7±3.2	
a* [a]	Baseline	14.7±3.7	0.7±1.2	14.7±1.8	0.6±2.9	15.5±2.5	-0.1±1.7	15.2±1.8	0.7±1.2
	6 weeks	15.3±3.2		15.3±3.8		15.4±2.3		15.9±1.7	
b*	Baseline	13.5±2.9	1.0±2.1	12.6±2.4	-0.1±2.4	14.3±1.9	0.5±1.9	14.5±2.8	-0.2±1.7
	6 weeks	12.5±2.8		12.5±2.7		14.7±1.9		14.3±2.4	
Gross Elasticity (%)	Baseline	75.3±5.8	-0.4±7.3	75.0±6.4	-0.4±10.3	65.1±10.4	0.2±15.1	62.4±6.5	8.5±7.7
	6 weeks	74.9±6.3		74.7±9.9		65.2±14.7		70.9±9.3#	

Net Elasticity[a] (%)	Baseline	55.1±9.8	-1.0±12.9	55.9±7.3	2.9±9.3	45.9±11.9	0.6±14.3	39.5±6.5	13.3± 13.2&
	6 weeks	54.1±10.3		58.7±10.1		46.5±14.7		52.7±12.6#	
Viscoelasticity	Baseline	28.3±8.1	-4.8±10.9	30.2±5.6	-4.2±7.8	30.3±7.2	-0.6±4.9	28.8±5.1	-1.1±5.5
	6 weeks	23.5±6.6		25.9±4.5		29.7±5.0		27.7±3.7	
Biological Elasticity (%)	Baseline	43.0±7.0	0.9± 8.6	43.0±6.4	2.7±9.9	33.2±6.9	3.9±9.4	30.3± 5.4	8.9±11.1
	6 weeks	43.8±8.6		45.8±9.3		37.1±11.4		39.1±12.2#	
Wrinkle[a]	Baseline	127.0±23.7	-8.5± 14.5	129.7±27.7	-4.4±16.5	103.5±21.2	-0.6±25.1	102.9±21.9	-10.9±7.4
	6 weeks	118.5±20.8		125.3±23.1		102.9±18.4		92.0±19.2#	
Smoothness	Baseline	286.1±79.1	-20.0± 43.1	267.7±63.3	14.3±36.8	298.8±102.6	-34.1 ±75.7	267.9±35.6	3.2±29.6
	6 weeks	266.1±54.5		282.0±75.7		264.7±45.4#		264.7±40.3	
Roughness[a]	Baseline	3.03±0.49	0.72±0.87	3.52±0.85	-0.13±0.97	2.70±0.74	0.15±1.12	2.49±0.73	0.19±0.66
	6 weeks	3.76±1.09		3.39±1.05		2.85±0.88		2.68±0.78	
Scaliness	Baseline	0.09±0.20	-0.06 ±0.18	0.04±0.10	0.05±0.14	0.35±0.58	-0.13 ±0.55	0.32±0.40	-0.13 ±0.25
	6 weeks	0.04±0.06		0.08±0.16		0.22±0.25		0.19±0.21	

Data are expressed as means± SD (n=11) and organized by time and treatment. [a] Log transformed for statistical comparison; # Significant difference between baseline and after six weeks in the same treatment groups by post hoc contrast. & Significant difference between change from baseline to final cranberry beverage and placebo by post-hoc linear contrast.

Table S6. Skin parameters on the forearm in women who consumed cranberry beverage or placebo for six weeks in participants stratified by age.

		<40 years old (n=11)				≥40 years old (n=11)			
Skin parameters	Time Period	Placebo	Change from baseline	Cranberry beverage	Change from baseline	Placebo	Change from baseline	Cranberry beverage	Change from baseline
TEWL (g/h/m^2)[a]	Baseline	8.1±4.3	0.3±5.0	7.7±3.6	-1.5±3.1	7.2±2.6	0.5±3.2	8.0±1.7	-1.2± 1.3
	6 weeks	8.4±7.9		6.2±1.3		7.7±2.6		6.8±2.0	
Hydration	Baseline	41.8±11.6	-0.1±13.4	37.7±6.6	0.2±7.5	36.4±8.7	5.8±6.9	39.8±8.6	3.2± 6.9
	6 weeks	41.7±9.0		37.9±6.8		42.2±8.7		42.9±8.9	
pH	Baseline	5.4±0.56	-0.02± 0.59	5.5±0.44	0.1±0.7	5.7±0.5	-0.01 ±0.56	5.7±0.5	0.1±0.3
	6 weeks	5.4±0.3		5.64±0.6		5.7±0.4		5.7±0.5	
Melanin Index[a]	Baseline	112.0±33.8	-6.1±17.0	124.3±40.6	-12.9 ±43.8	137.6±41.8	2.2±12.1	139.4±44.8	3.8± 14.1
	6 weeks	105.9±42.9		111.4±40.6		139.5±50.3		143.2±44.3	
Erythema Index	Baseline	183.2±49.9	-23.5 ±47.6	181.4±51.8	-6.4±39.5	214.8±44.1	17.6 ±28.2	225.5±53.2	16.2±21.4
	6 weeks	159.7±60.0		175.0±53.3		232.4±60.3		241.8±60.9	
L*[a]	Baseline	67.2±3.2	0.3±2.1	67.2±3.4	-0.1±2.1	63.2±3.9	-0.1±1.2	63.2±3.6	-0.8 ± 1.6
	6 weeks	67.5±4.1		67.1±3.6		63.1±3.9		62.4±4.31	
a*	Baseline	7.4±1.3	-0.01 ±0.86	7.3±1.0	0.5±0.5	8.6±1.1	0.3±0.8	9.1±1.1	0.1±0.9
	6 weeks	7.4±1.1		7.8±1.1		8.9±1.1		9.1±1.2	
b*	Baseline	12.7±3.8	0.3± 1.4	12.3±3.56	0.2±1.0	15.1±2.2	0.3±1.1	15.2±2.6	0.3±1.2
	6 weeks	13.0±3.5		12.5±3.6		15.4±2.2		15.5±2.8	

Gross Elasticity[a] (%)	Baseline	86.4±4.1	0.5±5.1	83.5±5.4	3.7±3.4	76.4±10.6	-1.7±8.2	72.8±10.7	7.1±7.8&
	6 weeks	86.9±4.6		87.1±4.7		74.8±13.4		79.9±7.6#,^	
Net Elasticity (%)	Baseline	86.1±8.1	-0.6±6.8	84.7±7.5	3.9±6.3	70.3±11.5	0.9±8.4	68.4±12.1	9.9±10.7&
	6 weeks	84.7±4.4		87.4±8.7		71.1±13.8		77.2±12.7#,^	
Viscoelasticity[a]	Baseline	28.5±5.9	-1.8±5.1	34.0±18.3	-7.7±18.9	31.5±4.7	2.1±7.6	34.8±10.4	-4.1±7.0
	6 weeks	26.7±7.4		26.3±5.6#		33.5±6.4		30.7±8.1	
Biological Elasticity (%)	Baseline	67.1±6.2	0.6±6.5	66.1±7.0	3.2±5.8	53.5±8.8	0.2±8.1	51.0± 12.1	5.7±9.0
	6 weeks	67.7±4.6		69.4±6.4		53.7±11.7		56.7±7.4#	
Wrinkle	Baseline	74.7±15.3	-2.9±22.3	73.0±20.9	-0.5±10.7	68.4±17.2	-2.9±9.2	69.0±19.7	-4.7± 8.8
	6 weeks	71.8±27.1		72.5±21.3		65.5±17.0		64.3±14.7	
Smoothness[a]	Baseline	208.1±26.4	-15.4 ± 42.5	219.6±47.0	-20.2 ± 47.8	227.8±45.5	3.1± 54.46	239.0±40.3	-11.9± 44.9
	6 weeks	192.7±30.9		199.3±29.8		230.9±68.9		227.1±60.8	
Roughness	Baseline	1.83±0.76	0.09±0.45	2.19±1.42	0.59±1.49	1.34±0.39	0.01± 0.45	1.45±0.66	0.28±0.88
	6 weeks	1.74±0.72		1.60±0.82		1.35±0.66		1.73±0.84	
Scaliness[a]	Baseline	0.08±0.11	0.04±0.12	0.06±0.10	-0.02± 0.10	0.26±0.24	-0.01 ±0.23	0.30±0.29	0.06±0.12
	6 weeks	0.12±0.19		0.04±0.03		0.25±0.25		0.24±0.32	

Data are expressed as means± SD (n=11) and organized by time and treatment. [a] Log transformed for statistical; ^Significant difference between placebo and cranberry beverage after six weeks by post hoc contrast. # Significant difference between baseline and after six weeks in the same treatment groups by post hoc contrast. & Significant difference between change from baseline to final cranberry beverage and placebo by post-hoc linear contrast.

Table S7. Summary of parameters for partial least square discriminant (PLS-DA) models describing the overall differences in skin lipidomic affected by cranberry beverage and placebo*.

Model	N[a]	R^2X (cum)[b]	R^2Y (cum)[b]	Q^2 (cum)[c]	p-value[d]	R2 intercept[e]	Q2 intercept[e]
Placebo *vs* cranberry beverage final	2	0.469	0.840	0.755	<0.001	0.373	-0.154
Placebo *vs* cranberry beverage final (<40 years old)	2	0.557	0.951	0.883	<0.001	0.427	-0.186
Placebo *vs* cranberry beverage final (≥40 years old)	2	0.482	0.910	0.595	<0.001	0.441	-0.176
Cranberry beverage baseline *vs* final	2	0.266	0.964	0.910	<0.001	0.373	-0.236
Cranberry beverage baseline vs final (<40 years old)	2	0.379	0.981	0.947	<0.001	0.387	-0.254
Cranberry beverage baseline vs final (≥40 years old)	2	0.51	0.955	0.904	<0.001	0.429	-0.286
Placebo baseline *vs* final	2	0.427	0.84	0.748	<0.001	0.368	-0.148
Placebo baseline *vs* final (<40 years old)	2	0.527	0.97	0.942	<0.001	0.407	-0.226
Placebo baseline *vs* final (≥40 years old)	2	0.497	0.929	0.854	<0.001	0.419	-0.249

*Models were built upon stratum corneum lipids. [a]Number of components, [b] R^2X and R^2Y are cumulative modeled variation in the X and Y matrix, respectively, [c] Q^2 is the cumulative predicted variation in the Y matrix, [d] Based on cross validated analysis of variation (CV-ANOVA) predictive residuals, [e] Based on permutation analysis.

References:

1. Sun, J.; Liang, F.; Bin, Y.; Li, P.; Duan, C. Screening non-colored phenolics in red wines using liquid chromatography/ultraviolet and mass spectrometry/mass spectrometry libraries. *Molecules* **2007**, *12*, 679–693, doi:10.3390/12030679.

2. Brown, P.N.; Shipley, P.R. Determination of anthocyanins in cranberry fruit and cranberry fruit products by high-performance liquid chromatography with ultraviolet detection: single-laboratory validation. *J. AOAC Int.* **2011**, *94*, 459–466.

3. Cunningham, D.G.; Vannozzi, S.; O'Shea, E.; Turk, R. Analysis and standardization of cranberry products. In *Quality management of nutraceuticals*; Ho, C.-T., Zheng, Q. Y., Eds.; ACS Symposium Series; American Chemical Society: Washington, DC, 2001; Vol. 803, pp. 151–166 ISBN 9780841218840.

4. Prior, R.L.; Gu, L. Occurrence and biological significance of proanthocyanidins in the American diet. *Phytochemistry* **2005**, *66*, 2264–2280, doi:10.1016/j.phytochem.2005.03.025.
